# Using co‐creation and multi‐criteria decision analysis to close service gaps for underserved populations

**DOI:** 10.1111/hex.12923

**Published:** 2019-06-11

**Authors:** Duncan Mortimer, Angelo Iezzi, Marissa Dickins, Georgina Johnstone, Judy Lowthian, Joanne Enticott, Rajna Ogrin

**Affiliations:** ^1^ Centre for Health Economics, Monash Business School Monash University Clayton Victoria Australia; ^2^ Bolton Clarke Research Institute Bolton Clarke Brisbane Queensland Australia; ^3^ Southern Synergy, Department of Psychiatry at Monash Health, Southern Clinical School Monash University Clayton Victoria Australia; ^4^ School of Public Health and Preventive Medicine Monash University Clayton Victoria Australia; ^5^ Department of General Practice, School of Primary and Allied Health Care Monash University Clayton Victoria Australia; ^6^ Austin Health Clinical School University of Melbourne Melbourne Victoria Australia

**Keywords:** co‐creation, multi‐criteria decision analysis, patient‐centred policy

## Abstract

**Background:**

Navigating treatment pathways remains a challenge for populations with complex needs due to bottlenecks, service gaps and access barriers. The application of novel methods may be required to identify and remedy such problems.

**Objective:**

To demonstrate a novel approach to identifying persistent service gaps, generating potential solutions and prioritizing action.

**Design:**

Co‐creation and multi‐criteria decision analysis in the context of a larger, mixed methods study.

**Setting and participants:**

Community‐dwelling sample of older women living alone (OWLA), residing in Melbourne, Australia (n = 13‐37). Convenience sample of (n = 11) representatives from providers and patient organizations.

**Interventions:**

Novel interventions co‐created to support health, well‐being and independence for OWLA and bridge missing links in pathways to care.

**Main outcome measures:**

Performance criteria, criterion weights , performance ratings, summary scores and ranks reflecting the relative value of interventions to OWLA.

**Results:**

The co‐creation process generated a list of ten interventions. Both OWLA and stakeholders considered a broad range of criteria when evaluating the relative merits of these ten interventions and a “Do Nothing” alternative. Combining criterion weights with performance ratings yielded a consistent set of high priority interventions, with “Handy Help,” “Volunteer Drivers” and “Exercise Buddies” most highly ranked by both OWLA and stakeholder samples.

**Discussion and conclusions:**

The present study described and demonstrated the use of multi‐criteria decision analysis to prioritize a set of novel interventions generated via a co‐creation process. Application of this approach can add community voice to the policy debate and begin to bridge the gap in service provision for underserved populations.

## 1 INTRODUCTION

Older women living alone (OWLA) may be burdened by complex care needs due to a constellation of co‐morbidities that may include cancer, cardio‐vascular disease, cognitive decline, incontinence, frailty, diabetes and their related complications.^1^, ^2^, ^3^ Complex needs are typically met with complex treatment pathways and a sequence of transitions from community‐based care to acute care to sub‐acute care.^4^ Bottlenecks, service gaps and access barriers may prevent or delay transitions and/or preclude a return to independent living.^4^

Bottlenecks, service gaps and access barriers may be particularly problematic for social and community services (such as short‐term home care, food and transportation services, or ongoing community‐based care). For example, discharge home from acute or sub‐acute care may not be possible if informal care from family members is not available and supply of affordable short‐term home care is limited.^4^, ^5^ Put simply, “complex needs are often at odds with health care systems designed to treat patients needing acute episodic care” (p2123).^6^

This has not gone unnoticed by policymakers and fundholders. In Australia, the development of existing programmes for promoting health independence was motivated by recognition of the challenges faced by older people.^4^, ^5^ Older people living alone were a particular concern, partly because their complex care needs “…require greater emphasis on care planning and coordination in order to navigate the transition (from health services) back to the community and remain there safely” (p3).^4^ In addition, non‐government organizations and local government authorities have developed social care programmes to help older people stay well and avoid or delay transition to residential care.^7^, ^8^

Despite the availability of such programmes, navigating treatment pathways remains a challenge for older people with complex needs. Around one in five high‐need older people in high‐income countries report care coordination problems.^6^ Additional financial pressures and logistical/psychological barriers may partly explain why older people *living alone* sometimes report lower levels of service utilization and worse patient care experiences than populations with comparable levels of need.^9^, ^10^, ^11^ Older *women* may be further disadvantaged by structural barriers to economic and social participation.^12^ This is not to exclude the fact that other populations with complex needs face barriers to participation and experience difficulties in navigating treatment pathways.^13^, ^14^ Here, OWLA represent one of many underserved populations that may benefit from a novel and person‐centred approach to identifying persistent service gaps, generating potential solutions and prioritizing action.

The present study sought to address the particular challenges faced by OWLA in accessing health, social and community services that are consistent with their needs and preferences. To engage OWLA and stakeholders in this task, we integrated co‐creation^15^ and multi‐criteria decision analysis (MCDA)^16^, ^17^ in a novel application of existing methods. The sections that follow (a) review existing methods and identify weaknesses that may be addressed by our approach to integrating co‐creation and MCDA, and (b) describe and demonstrate the use of our approach for identifying persistent service gaps, generating potential solutions and prioritizing action in an OWLA population. Results include a set of “high priority” strategies that were specifically co‐created to support the well‐being and independence of OWLA and bridge missing links in pathways to care. To inform future applications, we discuss several challenges we encountered in applying MCDA to the set of interventions generated by the co‐creation process. Despite these challenges, we conclude that the methods described here can add community voice to the policy debate and help close persistent service gaps for underserved populations.

## 2 BACKGROUND

A broad range of methods have previously been applied to (a) identify persistent service gaps, (b) generate potential solutions and/or (c) prioritize action. To identify persistent service gaps, mapping the number of relevant services or providers against the number of potential users for different catchment areas can help to identify locations, services or location/service combinations with potential shortages.^18^ Typically, this service mapping approach limits the set of potentially relevant services by first identifying all existing services in the relevant catchment areas and then applying exclusion criteria to remove services not currently offered to the population of interest.^18^ As a result, service mapping is not well suited to identifying service gaps for which there is no existing solution or that have arisen due to a mismatch between service design and the needs and preferences of potential users.

To identify service gaps not identified by service mapping, consumer engagement can provide a patient‐centred perspective^19^ and is increasingly recognized as an essential element in service design and quality improvement.^20^, ^21^, ^22^ Consumer engagement also has the potential to generate solutions and prioritize action.^20^ While consumer engagement can take many forms, one recent example of good practice in consumer engagement (a) surveyed perceptions of service quality in a representative sample of service users to prioritize broad areas for improvement, (b) convened a Consumer Action Group of hospital staff and consumer representatives to develop and implement quality improvements and (c) used feedback from subsequent iterations of the consumer survey to fine‐tune quality improvements and prioritize next steps.^19^ When prioritizing areas for improvement in this example, respondents to the consumer survey allocated a fixed number of “points” across their top five areas for improvement (eg improved parking, information, hospital catering) to indicate a relative preference for changes in one area over another. When developing potential quality improvements, the Consumer Action Group followed an adapted version of the Breakthrough Series model, with an emphasis on collaborative learning rather than co‐design or co‐creation. While this approach has the potential to identify service gaps for which there is no existing solution and to develop novel solutions that meet consumer needs and preferences, it may prove difficult to replicate these outcomes without a structured process for co‐creation and without a more detailed understanding of consumer preferences.

Integrating structured processes for co‐creation and preference elicitation into an overarching consumer engagement framework therefore offers a potential remedy for the weaknesses of some existing methods. With regard to preference elicitation, MCDA can provide a detailed understanding of consumer preferences, including a description of potentially relevant performance criteria (components of “value” to consumers) and the relative weight given to these criteria by different types of consumer. This type of information can be used and re‐used to predict how service improvements would be “valued” by existing users, potential users and partners/carers, helping decision makers to navigate the often complex trade‐offs required when setting health‐care priorities.^23^

With regard to co‐design or co‐creation of services, existing frameworks offer structured processes for generating novel improvements or interventions that are consistent with consumer needs and preferences.^15^, ^24^ For example, one such process^24^ was structured as a series of steps or phases: commencing with research and analysis “using qualitative methods… to gain deep insights into end users’ context, wants and needs” (p20); progressing to idea generation “typically led by the design team” (p21); idea testing and refinement “using paper‐ or experience‐prototyping” (p21); and concluding with evaluation and priority setting “to increase collective ownership of the outcome, and to encourage shared responsibility for progress” (p23). The use of this type of structured process is claimed^25^ to generate improvements that are “more likely to be fit for purpose, acceptable, valuable and enduring” (p406) than alternative approaches to service improvement or redesign.

While existing frameworks for co‐creation have clear strengths in identifying persistent service gaps and generating potential solutions, this is not the case when it comes to prioritizing action. To prioritize action, existing frameworks for co‐creation typically rely on global assessments of “importance” or “value”^26^ or simple tools to visualize the performance of potential improvements across just two or three dimensions.^24^, ^27^ Neither approach yields a sufficiently detailed description of consumer preferences for rational and transparent priority setting or for the purposes of predictive modelling. Priority setting mechanisms embedded within existing frameworks for co‐creation would therefore need to be repeated when additional improvements or new interventions come under consideration, and may be unsuitable to support funding decisions.

A mirror image of this trade‐off operates for MCDA. Multi‐criteria decision analysis delivers the detailed and durable understanding of consumer preferences that is missing from existing frameworks for co‐creation but lacks a structured process for generating novel service improvements or interventions that meet consumer needs and preferences. The complementary strengths of MCDA and co‐creation suggest that combining the two methods will deliver a framework for consumer engagement that carries all of the strengths and none of the weaknesses of one or other of these methods used in isolation.

## 3 METHODS

To provide an overarching framework for patient engagement, we adapted an existing framework for co‐creation,^15^ with MCDA employed to meet two of its six essential elements. Implementing the first four elements of the framework (listed below) generated ten co‐created strategies/interventions to support health, well‐being and independence for OWLA and bridge missing links in pathways to care:

1. Engage—establishing meaningful relationships with participants.
2. Plan—establishing goals with participants.
3. Explore—learning about experiences and identification of improvement ideas.
4. Develop—work with participants to turn ideas into improvements (ie strategies or interventions).

A detailed description of methods for generating our set of co‐created interventions has been reported elsewhere.^28^

The final two elements of the framework (listed below) were completed using MCDA to “decide” which of the ten co‐created strategies/interventions should be further developed and implemented, and “prepare for change”:

- Decide—choose which improvements to make, and how to make them (ie set priorities).
- Prepare for change—turn improvement ideas into sufficient information to prompt action by key decision makers.

While the “decide” element of the framework could be undertaken with a number of different objectives in mind and using a broad range of methods, we took a person‐centred approach and prioritized the co‐created strategies/interventions in line with their “value” to OWLA using MCDA. To “prepare for change,” the use of MCDA made two key contributions. First, it generated detailed information regarding performance criteria, criterion weights, performance ratings, summary scores and ranks reflecting the relative value of interventions to OWLA. Second, it provided a mechanism for reconciling client and stakeholder perspectives regarding which interventions should be considered “high priority.” The present paper describes and demonstrates application of MCDA to our set of co‐created interventions.

The interventions generated by the co‐creation process potentially differed on several performance criteria including effectiveness in improving health, well‐being and independence; out‐of‐pocket cost; location of service; and accessibility/availability. Moreover, there was potential for each of the co‐created interventions to do better on some criteria but worse on others so that it was not possible to clearly identify an intervention that performed “best” across all criteria. In such circumstances, the relative weight given to each of the criteria matters and MCDA can be particularly valuable.

Multi‐criteria decision analysis entails five steps: (a) identify the set of relevant interventions and the set of criteria against which interventions are to be evaluated, (b) quantify the relative importance of each of the criteria and express this information as “weights,” (c) specify a measurement scale for each criterion and evaluate the performance of each intervention on each of the criteria to obtain “performance ratings,” (d) combine weights with performance ratings to obtain a “score” (typically a weighted average) summarizing each intervention's performance across all criteria, and (e) rank order interventions based on their summary scores.^29^, ^30^ By reducing the complex task of priority setting to this series of relatively simple steps, MCDA can “help organize and distil the large amount of information required for effective and defensible decision‐making” (p128).^29^

In the present application, our MCDA exercise included two iterations of Step 2, with feedback and discussion between elicitation of first‐try and final criterion weights to promote deliberation and produce “better” weights.^31^ In other studies, MCDA has also included a Step 6 wherein the rank order implied by weighted averages has been validated or revised by key stakeholders during group discussion of criteria, weights, performance and priorities in a deliberative process.^30^ MCDA has previously been used to incorporate patient preferences into health‐care decision making for a wide range of diseases and interventions.^17^, ^23^ For the present study, an online decision support system, Annalisa v1.0 [Maldaba Ltd], was used to implement Steps 2‐5 of the MCDA exercise.^30^, ^32^ A detailed description of methods for each step of the MCDA process is provided in Appendix S1. The running sheet, script and materials for delivery of the MCDA exercise are provided in Appendix S2.

Ethical approval (encompassing both co‐creation and MCDA components of the study) was obtained from the Royal District Nursing Service (now known as Bolton Clarke) Human Research Ethics Committee (approval number 170003); local ethics and research governance procedures were subsequently completed at the study site (Monash University Human Research Ethics Committee approval number 2017‐8379‐8692).

## 4 RESULTS

### 4.1 Step 1—Identifying relevant interventions and performance criteria

The ten interventions generated by the co‐creation process (Table 1), plus a “Do Nothing” alternative, formed the set of relevant interventions for the MCDA exercise. Here, “Do Nothing” characterizes the set of services, programmes and interventions currently available to respondents (“do nothing extra” rather than the absence of intervention or services).

**Table 1:** Co‐created interventions

Label	Description
Mobility and ability	
“Handy Help”	Handy person to do small tasks such as change a lightbulb, replace batteries in smoke detector or flip mattress
“Post‐Op Stay”	Patient transfer to 3‐star hotel for overnight stay post day surgery or minor procedure, with nurse on duty
“Exercise Buddies”	Find‐a‐buddy service to match older women to an exercise buddy with similar needs and interests
Transport	
“Volunteer Drivers”	Volunteer drivers using private vehicle, with no restrictions on reason for travel (ie not just medical appointments) or time spent at activity
Social connections/participation	
“Good Neighbour” programme	Letter from local council or MP, encouraging people to connect or reconnect with older residents in their community
“Friendly Visitor” programme	Volunteer visiting programme, matching volunteers with older residents in the local community
Financial	
“Safe Boarder” matching & vetting	Central hub to match older residents with existing boarder and home‐share services
“Hour‐4‐Hour” barter	Central hub where older residents can exchange hours of work and skills (eg sewing, cooking, gardening) with other older people in the local community
Lack of knowledge	
“Shortcut to Services” information packs	Information pack with list of local services plus advice on when and how to access each service
“My Service Map” mail‐out	Mail‐out of local service map plus advice on when and how to access each service

To identify relevant performance criteria, semi‐structured interviews were conducted with 37 participants (mean age: 73 years, age range: 57‐94 years; owner‐occupier: 68% [25/37]; retirement village: 30% [11/37]). We identified 26 themes in interview scripts, not all of which could be characterized as criteria for distinguishing between preferred and less preferred interventions. For example, the theme of “marital life” was referenced 97 times by 57% (21/37) of participants but these references were predominantly descriptive of participants’ past marital life and transition to current living arrangements. Similarly, the theme of “retirement/work life” was referenced 99 times by 46% (17/37) respondents but these references were predominantly descriptive of participants’ past employment, health‐related barriers to employment and transition to retirement. Other themes of peripheral relevance to the task of identifying relevant criteria for evaluating alternative interventions included “volunteering/charity work,” “thoughts on living alone,” “thoughts on retirement villages,” “social services accessed,” “services accessed (other than social services)” and “current service gaps.”

To identify performance criteria of particular relevance to OWLA, we excluded themes of peripheral relevance and then ranked remaining themes by total references and by number of respondents referencing each theme. We then mapped themes to criteria suitable for evaluating alternative interventions. For example, general cost of living, cost of housing, household budgeting and out‐of‐pocket cost as a barrier to accessing health and social services were referenced 78 times by 57% (21/37) of respondents. We collected these references into a “finance” theme and mapped this theme directly to “Finances” in our list of potentially relevant criteria. In other cases, multiple themes could be mapped to the same criteria. For example, the themes of “support independence,” “transport” and “importance of living independently” were mapped to “being independent” in our list of potentially relevant criteria. This mapping is analogous to “bottom‐up” construction of the value tree, clustering characteristics of interventions into higher‐order objectives that are themselves sub‐components of overall value.^16^

Table 2 ranks potentially relevant themes by total references and number of respondents referencing each theme. Table 2 also serves to summarize our mapping from themes to potentially relevant criteria. After mapping themes to criteria, we were left with eight potentially relevant criteria, very close to the average number of criteria from a recent review^33^ of MCDA applications for health‐care decision making (mean = 8.2, range = 3‐19). Pilot tests of three versions of the MCDA exercise (Appendix S1) led us to omit one potentially relevant criterion (“Pet Friendly”), leaving us with a final set of seven criteria: “Health & Wellbeing,” “Relationships,” “Being Independent,” “Enjoyment,” “Finances,” “Accessible” and “Safety & Trust.”

**Table 2:**
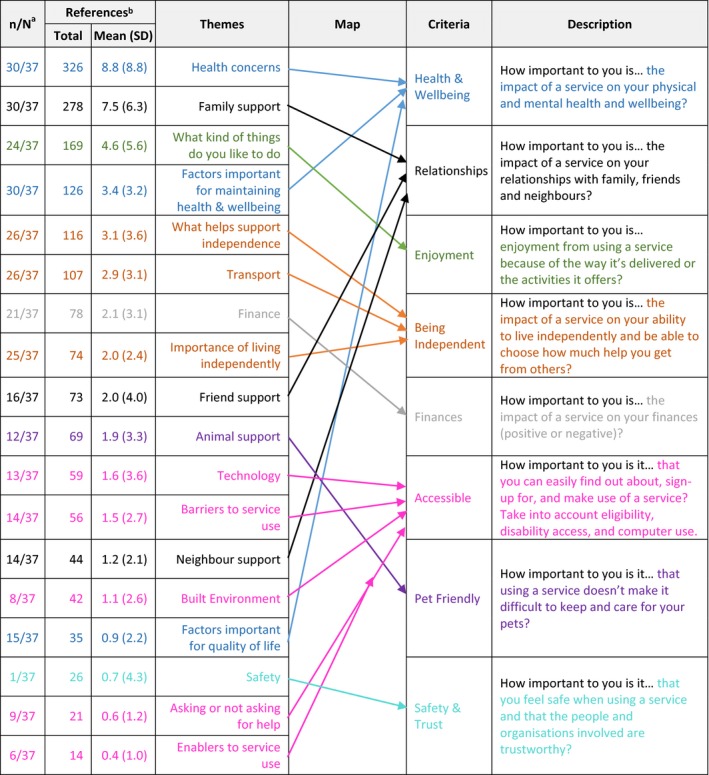
Mapping themes to criteria

^a^Number of respondents with at least one reference for the relevant theme.

^b^Total references for all n = 37 respondents and mean (SD) references per respondent.

### 4.2 Step 2—Weight criteria

Table 3 describes characteristics of the study sample in which we elicited criterion weights (Step 2) and performance ratings (Step 3). Table 4 describes “first‐try” and “final” criterion weights for OWLA and stakeholder participants. Appendices S1 and S2 provide a detailed description of methods for elicitation of “first‐try” and “final” criterion weights.

**Table 3:** Characteristics of the study sample for the elicitation exercises

Participant characteristics	OWLA (n = 13)	Stakeholder (n = 11)	Total (n = 24)
	n/N (%) or Mean (SD), Min‐Max		
Female	13/13 (100%)	11/11(100%)	24/24 (100%)
Age	72 (8.7), 56‐87	54 (12.9), 35‐73	64 (13.9), 35‐87
Born in Australia	10/13 (77%)	10/11 (91%)	20/24 (83%)
Education, highest level			
Primary	0/13 (0%)	0/11 (0%)	0/24 (0%)
Secondary	6/13 (46%)	1/11 (9%)	7/24 (29%)
University/post‐secondary	6/13 (46%)	3/11 (27%)	9/24 (38%)
Post‐graduate	1/13 (8%)	7/11 (64%)	8/24 (33%)
SEIFA Index by postcode, decile rank in Australia			
Socio‐economic disadvantage^a^	7.4 (2.6), 1‐10	8.0 (1.7), 4‐10	7.7 (2.2), 1‐10
Education and occupation^b^	7.5 (2.2), 3‐10	8.6 (1.4), 5‐10	8.0 (1.9), 3‐10

a The SEIFA Index of socio‐economic disadvantage (ABS, 2011) describes the economic and social disadvantage of individuals and households resident in a postcode area. Higher index scores indicate areas with a relative lack of disadvantage. The top (bottom) decile would be comprised of areas with the lowest (highest) level of disadvantage.

b The SEIFA Index of education and occupation (ABS, 2011) describes the education and occupation of individuals and households resident in a postcode area. Higher index scores indicate areas with many individuals with higher qualifications, employed in high‐skill occupations. The top (bottom) decile is comprised of areas with the highest (lowest) index scores.

**Table 4:** Criterion weights

Criteria	OWLA (n = 13)	Stakeholder (n = 11)	Total (n = 24)
	Mean weight% (SD), Min‐Max		
First‐try			
Health & wellbeing	24.5% (6.9), 13‐38	20.7% (9.9), 8‐35	22.8% (8.5), 8‐38
Relationships	6.0% (5.7), 0‐17	4.9% (4.8), 0‐14	5.5% (5.2), 0‐17
Enjoyment	7.1% (4.1), 0‐15	8.8% (7.3), 0‐27	7.8% (5.7), 0‐27
Being independent	22.7% (10.8), 10‐50	23.0% (15.2), 5‐51	22.9% (12.7), 5‐51
Finances	13.5% (8.1), 4‐30	10.4% (5.2), 3‐17	12.1% (6.9), 3‐30
Accessible	11.4% (4.4), 5‐19	12.2% (7.3), 0‐26	11.8% (5.8), 0‐26
Safety & trust	14.8% (11.5), 0‐37	20.1% (9.9), 4‐40	17.2% (10.9), 0‐40
Final			
Health & wellbeing	25.4% (6.4), 13‐39	25.5% (6.0), 18‐38	25.4% (6.1), 13‐39
Relationships	6.2% (5.9), 0‐18	5.7% (4.7), 0‐15	6.0% (5.2), 0‐18
Enjoyment	6.7% (4.6), 0‐16	6.9% (4.8), 0‐16	6.8% (4.6), 0‐16
Being independent	24.0% (10.6), 10‐53	23.5% (10.1), 4‐39	23.8% (10.1), 4‐53
Finances	13.2% (8.5), 0‐31	11.1% (4.8), 4‐17	12.3% (7.0), 0‐31
Accessible	10.7% (6.0), 2‐20	10.6% (5.9), 0‐18	10.6% (5.8), 0‐20
Safety & trust	13.7% (10.2), 0‐32	16.7% (7.3), 4‐27	15.1% (8.9), 0‐32

For OWLA participants, “Health & Wellbeing” carried the highest weight (most important), followed by “Being Independent,” “Safety & Trust,” “Finances,” “Accessible,” “Enjoyment” and “Relationships” (least important). Differences between OWLA and stakeholder weights were small and statistically insignificant for the first‐try elicitation (*t* ≤ 1.21, *P* ≥ 0.24). However, these small differences in importance weights still resulted in differences in importance rankings. For stakeholder participants, “Being Independent” carried the highest weight (most important), followed by “Health & Wellbeing,” “Safety & Trust,” “Accessible,” “Finances,” “Enjoyment” and “Relationships” (least important). The minor differences between OWLA and stakeholder weights were further reduced after deliberation, with the closer correspondence in final weights due mostly to a shift in stakeholder weights. This closer correspondence in final weights was also seen in importance rankings, with OWLA and stakeholder importance rankings based on final weights matching importance rankings based on first‐try OWLA weights. When movements in OWLA and stakeholder weights were pooled, the largest changes in weight between first‐try and final elicitations were observed for “Health & Wellbeing” (+2.6%) and “Safety & Trust” (−2.1%).

### 4.3 Step 3—Rate performance on each criterion

Table 5 describes performance ratings for each intervention against each criterion. On average, “Do Nothing” was rated lowest across all criteria by both OWLA and stakeholders, largely as a consequence of the fact that co‐created interventions “added something” to the “Do Nothing” alternative. For positively valued criteria, “adding something” might plausibly result in better performance, provided that the relevant intervention “does no harm” with respect to the relevant criteria. Neither OWLA nor stakeholder samples identified a dominant intervention that performed best across all criteria. For OWLA, “Handy Help” received the highest mean rating on “Health & Wellbeing,” “Finances,” “Being Independent,” “Accessible” and “Safety & Trust” but “Good Neighbour” received the highest mean performance rating on “Relationships” and “Exercise Buddies” received the highest mean rating on “Enjoyment.” For stakeholders, “Exercise Buddies” received the highest mean rating on “Health & Wellbeing,” “Enjoyment” and “Relationships” but “Handy Help” received the highest mean rating on “Finances,” “Being Independent” and “Safety & Trust.”

**Table 5:** Performance ratings

Criteria/Interventions	Health & wellbeing	Relationships	Enjoyment	Being independent	Finances	Accessible	Safety & trust
OWLA (n = 13), mean rating% (SD)							
“Do Nothing”	0% (0)	8% (28)	8% (28)	0% (0)	8% (28)	0% (0)	0% (0)
Handy Help	77% (33)	43% (33)	39% (32)	76% (38)	80% (27)	63% (35)	69% (28)
Post‐Op Stay	49% (34)	32% (35)	30% (30)	54% (33)	50% (31)	54% (26)	58% (22)
Exercise Buddies	50% (29)	52% (30)	71% (30)	61% (34)	40% (33)	49% (30)	46% (24)
Volunteer Drivers	48% (29)	55% (32)	57% (36)	71% (33)	51% (33)	61% (34)	69% (26)
Good Neighbour	41% (27)	73% (31)	55% (34)	50% (22)	49% (37)	42% (35)	50% (30)
Friendly Visitor	33% (28)	72% (32)	60% (33)	42% (32)	45% (34)	42% (33)	49% (33)
Safe Boarder	18% (19)	42% (33)	32% (30)	35% (32)	61% (37)	41% (32)	68% (40)
Hour‐4‐Hour barter	35% (25)	44% (29)	48% (31)	56% (34)	64% (32)	48% (32)	29% (18)
Shortcut to Services	43% (32)	28% (29)	29% (28)	47% (36)	38% (21)	56% (39)	47% (32)
My Service Map	48% (36)	32% (34)	42% (36)	47% (40)	38% (32)	68% (43)	46% (39)
Stakeholders (n = 10)^a^, mean rating% (SD)							
“Do Nothing”	0% (0)	0% (0)	0% (0)	0% (0)	0% (0)	0% (0)	0% (0)
Handy Help	52% (37)	35% (37)	29% (33)	79% (24)	73% (32)	68% (32)	81% (29)
Post‐Op Stay	59% (34)	27% (38)	18% (27)	35% (36)	38% (30)	46% (41)	47% (36)
Exercise Buddies	77% (28)	63% (32)	85% (26)	48% (35)	26% (28)	51% (30)	47% (32)
Volunteer Drivers	56% (30)	48% (33)	41% (36)	77% (35)	70% (26)	69% (40)	61% (43)
Good Neighbour	25% (27)	38% (36)	45% (37)	31% (32)	23% (31)	31% (36)	56% (38)
Friendly Visitor	50% (26)	60% (29)	54% (38)	28% (29)	30% (36)	37% (33)	68% (35)
Safe Boarder	15% (13)	33% (32)	30% (29)	29% (29)	56% (38)	27% (25)	75% (37)
Hour‐4‐Hour barter	33% (23)	50% (39)	54% (25)	32% (27)	42% (32)	28% (30)	31% (25)
Shortcut to Services	36% (29)	20% (35)	13% (16)	48% (34)	22% (23)	48% (37)	25% (29)
My Service Map	50% (41)	22% (34)	9% (15)	48% (37)	18% (26)	45% (39)	25% (31)
Total (n = 23), mean rating% (SD)							
“Do Nothing”	0% (0)	4% (21)	4% (21)	0% (0)	4% (21)	0% (0)	0% (0)
Handy Help	66% (36)	40% (34)	35% (32)	78% (32)	77% (29)	65% (33)	74% (28)
Post‐Op Stay	54% (34)	30% (36)	25% (28)	46% (35)	45% (30)	51% (33)	54% (29)
Exercise Buddies	62% (31)	57% (31)	77% (29)	55% (35)	34% (31)	50% (29)	47% (27)
Volunteer Drivers	52% (29)	52% (32)	50% (36)	74% (33)	59% (31)	64% (36)	65% (34)
Good Neighbour	34% (28)	58% (37)	51% (35)	42% (28)	38% (37)	37% (35)	53% (33)
Friendly Visitor	40% (30)	67% (31)	57% (34)	36% (31)	38% (35)	40% (33)	57% (34)
Safe Boarder	17% (16)	38% (32)	31% (29)	33% (30)	58% (37)	35% (29)	71% (38)
Hour‐4‐Hour barter	34% (23)	47% (33)	50% (28)	46% (33)	54% (33)	39% (32)	30% (21)
Shortcut to Services	40% (30)	25% (31)	22% (24)	47% (34)	31% (23)	53% (38)	37% (32)
My Service Map	49% (37)	28% (34)	28% (33)	47% (38)	30% (31)	58% (42)	37% (37)

a One respondent lost to follow‐up after completing first‐try and final criteria weighting but before completion of performance rating.

### 4.4 Steps 4 and 5—Summarize performance across all criteria and rank from “best” to “worst”

Table 6 summarizes scores and ranks for our set of interventions. Differences between OWLA and stakeholders with respect to first‐try criterion weights and with respect to performance ratings were carried through and reflected in differences on intervention scores and ranks. When intervention scores were calculated from OWLA data, the most preferred intervention was “Handy Help,” followed by “Volunteer Drivers,” “Post‐Op Stay,” “Exercise Buddies,” “Good Neighbour,” “MyServiceMap,” “Hour‐4‐Hour Barter,” “Shortcut to Services,” “Friendly Visitor,” “Safe Boarder” and “Do Nothing” (least preferred). The top two interventions remained the same when intervention scores were calculated from stakeholder data but we see “Post‐Op Stay” drop to fifth position and a reordering of lower ranked interventions.

**Table 6:** Intervention scores and ranks

Interventions	OWLA (n = 13)	Stakeholder (n = 11)^a^	Total (n = 24)
	Using first‐try weights, mean score% (SD); rank of means		
“Do Nothing”	2% (5); 11th	0% (0); 11th	1% (4); 11th
Handy Help	70% (27); 1st	66% (23); 1st	68% (25); 1st
Post‐Op Stay	53% (23); 4th	40% (24); 5th	47% (24); 4th
Exercise Buddies	52% (23); 3rd	56% (22); 3rd	54% (22); 3rd
Volunteer Drivers	60% (24); 2nd	61% (26); 2nd	61% (26); 2nd
Good Neighbour	51% (23); 5th	34% (25); 10th	43% (25); 7th
Friendly Visitor	43% (24); 9th	46% (24); 4th	45% (24); 5th
Safe Boarder	41% (24); 10th	37% (23); 6th	39% (23); 10th
Hour‐4‐Hour barter	46% (26); 8th	34% (23); 9th	41% (23); 8th
Shortcut to Services	45% (29); 7th	35% (26); 8th	40% (26); 9th
My Service Map	48% (31); 6th	37% (29); 7th	43% (29); 6th
	Using final weights, mean score% (SD); rank of means		
“Do Nothing”	1% (5); 11th	0% (0); 11th	1% (3); 11th
Handy Help	71% (27); 1st	63% (21); 1st	67% (24); 1st
Post‐Op Stay	52% (23); 4th	41% (26); 5th	47% (24); 4th
Exercise Buddies	53% (23); 3rd	57% (20); 3rd	55% (21); 3rd
Volunteer Drivers	61% (24); 2nd	62% (29); 2nd	61% (25); 2nd
Good Neighbour	51% (22); 5th	32% (24); 10th	42% (24); 7th
Friendly Visitor	43% (24); 9th	45% (25); 4th	44% (24); 6th
Safe Boarder	41% (24); 10th	36% (22); 7th	39% (23); 10th
Hour‐4‐Hour barter	47% (26); 7th	34% (17); 9th	41% (23); 8th
Shortcut to Services	46% (29); 8th	35% (20); 8th	41% (25); 9th
My Service Map	48% (31); 6th	39% (27); 6th	44% (29); 5th

a Calculation of scores and ranks was based on each participant's criterion weights and performance ratings, except for n = 1 from the stakeholder group where missing data for performance ratings were replaced with mean performance ratings for stakeholder participants.

The closer correspondence between OWLA and stakeholders on final weights also resulted in a closer correspondence in intervention scores and ranks. When intervention scores were calculated using final weights, the three most highly ranked interventions were the same for OWLA and stakeholder samples. The lack of consistency between OWLA and stakeholder rankings of lower ranked interventions persisted even after replacing first‐try weights with final weights.

## 5 DISCUSSION

### 5.1 Principal findings and innovations

The present study demonstrated a novel application of co‐creation^15^ and MCDA^16^, ^17^ for identifying and closing persistent service gaps. Results suggest that OWLA continue to face service gaps and/or barriers to accessing existing services despite the introduction of government programmes designed to facilitate and coordinate care for patients with complex needs. OWLA identified limitations of existing services in areas of transportation, meals preparation and delivery, home help, social and leisure activities, nursing and ambulatory care, social visiting (including pet‐visiting services) and companionship, sub‐letting and home‐share, paid and unpaid carers, and employment/volunteering opportunities. To address limitations of existing services, OWLA co‐created ten interventions and described these in sufficient detail for the purposes of evaluation and priority setting (including details such as limits on eligibility, out‐of‐pocket cost and accessibility).

Results also confirmed that OWLA and stakeholders consider a broad range of criteria when evaluating the relative merits of different interventions. Both OWLA and stakeholders assigned weight to criteria that are not routinely captured in standard incremental cost‐effectiveness ratios. Combining criterion weights with performance ratings (to calculate scores and ranks) yielded a consistent set of high priority interventions, with “Handy Help,” “Volunteer Drivers” and “Exercise Buddies” most highly ranked by both OWLA and stakeholder samples.

These results are partly a function of methodological decisions designed to tailor co‐creation and MCDA to our purpose. Given (a) the continued presence of service gaps^6^ and (b) the importance of psychological barriers to care for OWLA,^9^ we emphasized OWLA preferences at each step of the co‐creation and MCDA processes. In previous applications of MCDA, criterion weights have sometimes been derived using relatively complex quantitative methods such as discrete choice experiments (DCEs).^29^ Some studies have flagged potential difficulties in using this approach to obtain weights from community members.^16^ For example, Youngkong et al^30^ reported that their sample of people living with HIV/AIDS “had difficulties in completing the DCE survey and interpreting DCE findings” (p6).^30^ To minimize barriers to participation, we opted for direct elicitation of weights and performance ratings via a user‐friendly graphical interface^32^ and providing almost one‐to‐one support for OWLA participants. In other applications of MCDA, much simpler methods have been employed to “weight” priority setting criteria. For example, Goetghebeur et al^34^ directly elicited criterion weights from a panel of key stakeholders, with possible weights ranging from 1 (lowest) to 5 (highest) and nothing to preclude respondents from attaching the highest (or lowest) weight to all criteria. To ensure weights reflected the relative importance of performance criteria, we constrained the sum of weights to 100% and included checks for internal consistency.

### 5.2 Limitations

The present study offers a number of lessons for researchers and policymakers. First, the co‐creation process yielded a set of 10 interventions, designed in collaboration with OWLA participants to close service gaps and/or to remove barriers to accessing existing services. Given that these interventions were co‐created to meet previously unmet needs, we could not rely on a well‐developed evidence base to describe performance across each of our seven performance criteria. We instead populated the performance matrix (Step 3 of the MCDA exercise) based on the expert opinion of stakeholders and OWLA participants. Further research may therefore be required to update the performance matrix and to re‐calculate scores and ranks as additional evidence becomes available. More generally, further thought should be given to resolving the tension between giving voice to relevant communities and evidence‐based decision making; that is to say, how to design person‐centred care in the absence of high‐quality evidence on person‐relevant outcomes?

Second, our sample for the elicitation tasks (Steps 2 and 3 of the MCDA exercise) included a diverse cross‐section of OWLA and broad representation of providers and patient organizations. Older women living alone were drawn from postcode areas from the full span of socio‐economic disadvantage (lowest decile and highest decile) and included younger and older women within the OWLA population (range: 56‐87 years). Stakeholder participants were drawn from a wide range of providers and patient organizations including local government, service providers, charitable organizations, women's advocacy groups and various relevant peak bodies. However, all of the provider and patient organizations represented in the stakeholder sample were located in metropolitan Melbourne. For the OWLA sample, the majority of participants were highly educated and only a small proportion of participants were drawn from culturally and linguistically diverse (CALD) populations or from the growing group of OWLA over 85 years of age. Where needs, preferences and barriers to access are specific to population sub‐groups (eg defined by CALD, older age, educational attainment or geographic location), the exercise described here may be need to be repeated to identify and close persistent service gaps incident upon those populations.

Third, while our sampling strategy afforded access to a broad range of perspectives, practical and financial constraints on sample size left us underpowered for statistical tests of sub‐group differences and between‐intervention or between‐criteria differences in weights, ratings and scores. Of particular note and despite our use of a simplified and user‐friendly implementation of MCDA, many OWLA participants required in‐person demonstrations of how to complete elicitation tasks and one‐on‐one computing assistance to access and navigate the decision support software. The level of support required suggests that—for an OWLA population—postal or remote online administration of an MCDA exercise is unlikely to prove fruitful. Researchers considering application of our approach in other underserved populations may encounter similar barriers but should give due consideration to online administration in a larger sample if levels of computer literacy and education permit.

Finally, feedback and discussion between elicitation of “first‐try” and “final” criterion weights were designed to promote deliberation and with the intent of producing “better” weights. For unfamiliar or complex trade‐offs, preferences (eg as reflected in criterion weights) may not be fully formed or readily accessible and may instead need to be “constructed” by respondents. Deliberation can help in this construction process, affording time and space for careful consideration of new information, alternative viewpoints and the respondent's own values. Promoting deliberation in valuation or elicitation tasks can therefore get us closer to an understanding of each respondent's *true* preferences.^31^ A small number of studies have evaluated the impact of methodological choices in implementation of citizen's juries, deliberative focus groups or citizen's panels, including varying the number of participants, varying the duration of individual sessions and of the overall process, providing access to content experts and face‐to‐face vs telephone interaction.^35^ As yet, these studies provide no clear guidance on the impact of longer vs shorter deliberation, or repeated vs one‐time interaction. In the present study, differences between “first‐try” and “final” weights were small in magnitude and it is unlikely that additional iterations of elicitation and deliberation would have resulted in large‐scale deviations from our “final” weights. Nonetheless, the results reported here may in part reflect our methodological choices and future applications may find value in allowing additional iterations of the elicitation task or more time for deliberation.

## 6 CONCLUSION

The present study described and demonstrated a novel approach to integrating co‐creation and MCDA. Results suggest that OWLA continue to encounter service gaps and barriers to service use. This is despite the introduction of government programmes designed to facilitate and coordinate care for patients with complex needs. Results also suggest that giving voice to community preferences via the approach described here may help to close persistent service gaps and lower access barriers. Further work is underway to ensure that these community preferences are given due to weight by decision makers.

## CONFLICT OF INTEREST

None.

## ETHICAL APPROVAL

Ethical approval (encompassing both co‐creation and MCDA components of the study) was obtained from the Royal District Nursing Service Human Research Ethics Committee (approval number 170003); local ethics and research governance procedures were subsequently completed at the study site (Monash University Human Research Ethics Committee approval number 2017‐8379‐8692).

## Data Availability

The data that support the findings of this study are available on request from the corresponding author. The data are not publicly available due to privacy or ethical restrictions.
