# Supplementary material for: Using co‐creation and multi‐criteria decision analysis to close service gaps for underserved populations
Source: Health Expect. 2019 Jun 11;22(5):1058–68. doi: 10.1111/hex.12923 (PMC6803401; doi:10.1111/hex.12923)
Supplement: Supplementary file 1 [file HEX-22-1058-s001.docx]

**Appendix: S1 – DETAILED METHODS FOR MCDA**

Multi-criteria decision analysis (MCDA) is necessarily specific to the decision problem at hand. Here, the decision problem was defined with the following considerations in mind. First, given the large number of relevant strategies / interventions and the reality of funding constraints, the project team considered it unlikely that all strategies / interventions would be implemented. Second, given the fragmented nature of funding and decision-making authority for OWLA, the project team considered it unlikely that the decision problem could be framed from the perspective of a single provider or fund-holder. The decision problem was therefore framed from the perspective of OWLA as decision-maker. Third, given the emphasis on unmet need and service gaps (and the failure of providers and funders to meet these needs and close these gaps), criteria for prioritising interventions were defined in line with OWLA objectives rather than provider / funder objectives. Taken together, the above considerations can be summarised in the following decision problem: which of the co-created strategies / interventions offer the highest ‘value’ to OWLA? The decision problem was presented to the project advisory group: the Stakeholder Group of Experts (SAGE) with membership comprising researchers, service providers and patient representatives, for comment. We tailored methods for each step of the MCDA exercise to the decision problem specified above.

***Step 1 – Identifying relevant interventions & performance criteria***

The ten interventions generated by the co-creation process, plus a ‘Do Nothing’ alternative, formed the set of relevant interventions for the MCDA exercise. For the purposes of identifying relevant performance criteria, semi-structured interviews were conducted with a stratified purposive sample of OWLA with strata defined by age, membership of culturally and linguistically diverse (CALD) groups, socio-economic advantage / disadvantage, education level, and geographic location. Interviews were structured to explore the lived experience of older women living alone; with a particular emphasis on predictors of vulnerability and resilience, use of existing health and community services, perceived enablers and barriers to accessing existing services, perceived service gaps, and characteristics of preferred (and non-preferred) services. Thematic analysis identified 26 themes in the interview scripts, not all of which could be characterised as criteria for distinguishing between preferred and less preferred interventions. For the purposes of identifying criteria of particular relevance to OWLA, we excluded themes of peripheral relevance (see main manuscript for further details) and then ranked remaining themes by total references and by number of respondents referencing each theme. We then mapped themes to criteria suitable for evaluating the relative merits of alternative interventions. After mapping themes to criteria, we were left with eight potentially relevant criteria: ‘Health & Wellbeing’, ‘Relationships’, ‘Being Independent’, ‘Enjoyment’, ‘Finances, ‘Accessible’, ‘Pet Friendly’, and ‘Safety & Trust’.

Community member participants in previous applications of MCDA have sometimes found it difficult to choose between alternative uses of resources; resulting in equal priority being given to all interventions.^1^ This may be partly attributable to the nature of the performance criteria. Specifically, criteria are typically dictated by the decision-making body and relate to coarse-grained characteristics of the intervention and target population such as system-level cost, average effectiveness, quality of evidence, and patient age, gender or severity.^1,2^ In contrast, we adopted a patient-centred approach; using the qualitative interviews to identify priority setting criteria of particular relevance to OWLA. While it was hoped that this approach would result in a clearer set of priorities, it carried the additional benefit of providing a more detailed and disaggregated understanding of community preferences.

In finalising the set of criteria for inclusion in the analysis, we applied four principles summarised in Marsh et al^3^: completeness (all relevant criteria included), non-redundancy (all irrelevant or unimportant criteria excluded), non-overlap (criteria should not overlap), and preference independence (no interactions between criteria). For the purposes of decision-making from a government or societal perspective (e.g. allocating health funding, commissioning research), additional criteria (such as cost to the public sector and distribution of benefits by age and socio-economic status) are likely to influence intervention scores / rankings. However, the present MCDA exercise was designed with a different objective in mind; to inform decisions regarding further development and evaluation of novel interventions generated via the co-creation process. The aim of this co-creation process was to identify and close service gaps that may reflect a mismatch between OWLA preferences and service design or a failure to take account of OWLA preferences in prioritising alternative interventions / services. Given these aims and objectives, we adopted a patient- or client-centred approach and limited the set of potentially relevant criteria to those identified by OWLA from an OWLA perspective.

While ‘completeness’ is relatively easy to achieve for our relatively narrow decision-problem, we may still face a trade-off between completeness / complexity of our MCDA and the validity of weights and scores elicited from OWLA respondents.^3,4^ We piloted three versions of the MCDA exercise with members of the extended project team to assess the likely magnitude of gains / losses in validity of weights and scores as we added / subtracted criteria. The first version included all eight criteria (‘Health & Wellbeing’, ‘Relationships’, ‘Being Independent’, ‘Enjoyment’, ‘Finances, ‘Accessible’, ‘Pet Friendly’, and ‘Safety & Trust’). The second version included the top six criteria when ranked by total references and by number of respondents referencing the underlying themes (‘Health & Wellbeing’, ‘Relationships’, ‘Being Independent’, ‘Enjoyment’, ‘Finances, and ‘Accessible’). The third version included the top five criteria (‘Health & Wellbeing’, ‘Relationships’, ‘Being Independent’, ‘Enjoyment’, and ‘Finances’).

All three versions were adjudged by the project team to be consistent with principles of non-redundancy and preference independence but further analysis of the qualitative data from our original semi-structured interviews suggested the potential for overlap between ‘Enjoyment’ and ‘Relationships’. While OWLA frequently referenced the importance of ‘Enjoyment’ in uptake of health and social services, some OWLA characterised ‘Enjoyment’ in terms of opportunities for social interaction (hence the potential for overlap with ‘Relationships’). We sought to minimise overlap between these two potentially relevant criteria by providing clarification and further information in our criteria descriptions and directing respondents to carefully read each of the criteria descriptions. During piloting, we requested feedback on the likelihood of overlap between ‘Enjoyment’ and ‘Relationships’ and for respondents to identify the potential for overlap between other criteria. Respondents confirmed that they were able to separate ‘Enjoyment’ from ‘Relationships’ and that their weight for ‘Enjoyment’ was not a function of the importance of ‘Relationships’ (or vice versa). Some respondents noted the potential for overlap between ‘Accessible’ and ‘Enjoyment’ or ‘Safety & Trust’ but that the potential for double-counting was low and advised against combining criteria into broader components of value.

Piloting suggested that fewer criteria would reduce complexity and reduce respondent burden. However, piloting also suggested the presence of a trade-off between complexity and completeness for the majority of criteria; with respondents recommending retention of criteria that mapped to rarely mentioned themes in the qualitative data such as ‘Safety & Trust’ and ‘Accessible’. For ‘Pet friendly’, discussions during piloting suggested that excluding this criterion would entail a minimal loss in terms of completeness; at least for the set of interventions to be evaluated in the present study. Note that this would not have been the case if our set of relevant interventions had included services such as pet-friendly residential care and, for some interventions such as service / assistance animal programs, the notion of ‘pet-friendliness’ may be insufficient to capture related aspects of ‘value’. Potentially relevant criteria were presented to OWLA participants and stakeholders during the first two co-creation forums for comment. Discussion during these two forums confirmed the importance of ‘Safety & Trust’ and ‘Accessible’, and confirmed the relatively low weight attached to ‘Pet Friendly’. For this reason, the final version of our MCDA exercise omitted ‘Pet Friendly’ and included the seven remaining criteria: ‘Health & Wellbeing’, ‘Relationships’, ‘Being Independent’, ‘Enjoyment’, ‘Finances, ‘Accessible’, and ‘Safety & Trust’.

***Step 2 – Weight criteria***

Performance criteria and the set of relevant interventions / strategies derived from Step 1 of the MCDA exercise were pre-populated into electronic forms for the purposes of eliciting criteria weights from OWLA and stakeholders. Specifically, we adapted an *Annalisa v1.0* [Maldaba Ltd, London] MCDA template (see <http://www.cafeannalisa.org.uk>) and embedded this template into an online questionnaire compiled and hosted using *Elicia* [Maldaba Ltd, London] (see <http://www.elicia.org.uk>). Electronic forms were used for data collection during the elicitation exercise, accessed via the *Elicia* website.

For the purposes of eliciting criteria weights (Step 2) and performance ratings (Step 3), members of the stratified purposive sample of OWLA who participated in the semi-structured interviews (Step 1) were invited to participate in an elicitation exercise (1.5-2 hours duration). Additional OWLA participants were identified by stratified purposive sampling with strata defined by geographic location. This sample selection strategy yielded a diverse sample of OWLA for the elicitation exercises, complemented by a convenience sample of stakeholder participants (representatives from providers and patient organisations). In order to facilitate the widest possible participation from OWLA and stakeholders, the MCDA exercise was delivered as four small group sessions. We held one small group session in the Melbourne CBD (n=5 OWLA and n=6 stakeholders) and three at inner suburban locations convenient to participants (one with n=5 stakeholders and two smaller group sessions with n=2 OWLA each). We also arranged one-on-one sessions at an inner suburban location or at the participant’s place of residence (or nominated venue) to facilitate participation where timing, logistics or preferences precluded attendance at group sessions (n=4 OWLA).

For elicitation during small group sessions, the elicitation exercise was moderated by a member of the research team (DM) with experience in moderating focus groups and stakeholder forums. Other members of the research team assisted participants in an almost one-to-one participant / assistant ratio. For one-on-one sessions, the elicitation exercise was moderated by MD or GJ; each of whom had already provided assistance with delivery of the elicitation exercise in the initial small group session. All sessions were moderated following a script written specifically for this exercise (see Supplementary Materials S2). Each session commenced with an overview of objectives, before proceeding to a moderated group discussion of performance criteria for a familiar consumer durable (domestic refrigerator) and a brief audio-visual presentation to demonstrate the potential importance of criteria weights in purchasing decisions. Participants were then provided with a hand-out summarising labels and descriptions for the set of performance criteria identified during Step 1 of the MCDA exercise (see Supplementary Materials S2); followed by moderated group discussion to clarify terms and familiarise participants with performance criteria for the co-created interventions.

At this point, participants were instructed to access the electronic forms for the elicitation exercise and to spend some time mapping from the labels / descriptions on the hand-out to the criteria labels in the electronic forms. Two sets of criteria weights were elicited for each participant: ‘first-try’ and ‘final’ weights, with restrictions and constraints specified in each case so that weights had a possible range from 0% (lowest possible weight) to 100% (highest possible weight) but the sum of weights across all criteria equalled 100%.

Participants were asked to complete the elicitation exercise for their first-try criteria weights “without discussing their views with anyone else”. After completing the elicitation exercise to obtain these first-try weights, one of the research team (AI) facilitated a short discussion of participants’ ranking and weighting of criteria while DM calculated group averages for OWLA participants and stakeholder participants. These group averages were presented to participants, followed by moderated discussion of differences between average and individual weights, differences between OWLA and stakeholder weights, and reasons for any such differences. Finally, participants were asked to consider whether they would like to make changes to their first-try weights but only to make changes “if the weightings from your first try don’t reflect what you think now after hearing the discussion and seeing our results”.

To elicit first-try weights, participants were instructed to first identify the most important criteria in assessing the value of interventions to OWLA and to assign a weight of 100% to this criteria. Participants were then asked to identify the second most important criteria and to assign a weight to reflect the relative importance of their first most important and second most important criteria (with the sum of weights for the first two criteria constrained to equal 100%). Respondents repeated this process for the remaining performance criteria, assigning progressively smaller weights to each criteria as they identified the next most important criteria from the pre-populated list. At several points in this process, participants were prompted to check for internal consistency; remembering that the weights for two criteria should not be the same unless they carry the same importance, that more important criteria should be assigned greater weight, and that the ratio of criteria weights should reflect the relative importance of different criteria. To elicit final weights, participants were asked to consider revisions to criteria weights; using their first-try weights as a starting point.

For the elicitation exercise described above, weights were to reflect the relative importance of the pre-populated performance criteria *in evaluating the value of interventions to OWLA*. OWLA and stakeholders were therefore instructed to complete the elicitation exercise from slightly different perspectives. Whereas OWLA participants were asked to respond based on their own preferences as potential consumers of the co-created interventions, stakeholders were asked to do their best to place themselves in the shoes of the older women that their organisation helps.

***Step 3 – Rate performance on each criteria***

A systematic literature review was conducted during the initial stages of the broader project. The aim of the systematic review was to summarise the available evidence regarding the effects of interventions designed to improve the health and wellbeing of older people (aged 55 years or more) living alone.^5^ Inclusion criteria were widened to include older men as well as women because OWLA populations are under-researched^6^, and because strategies/interventions may work across gender boundaries. Further detail regarding methods and results of the systematic review are reported elsewhere.

Results from the systematic review were to provide an evidence-base for populating the MCDA performance matrix; where the performance matrix comprises performance ratings for each intervention against each criteria. However, this first-best approach was commenced with the understanding that we may find little or no evidence for performance against one or more performance criteria; largely because our interventions were co-created to meet previously unmet needs. In addition to a sparse evidence-base for the novel interventions that emerged from our co-creation process, our patient-centred approach to defining value and identifying criteria left us with the further challenge of assessing performance against criteria that were important to OWLA but that were unlikely to have been captured in the outcome measures specified for intervention studies.

Where evidence could be identified regarding the effect of interventions for older men or women living alone, the interventions in question typically had no direct parallel in our set of co-created interventions. Evidence specific to older people living alone was available for just one of our co-created interventions: the friendly visitor program, though this evidence^7-9^ offered little guidance in populating our performance matrix. Andrews et al^7^ conducted semi-structured interviews with existing users of a local home-visiting befriending service for frail and isolated older people in the United Kingdom. While the qualitative approach employed by Andrews et al^7^ allowed detailed exploration of positive and negative opinions of the service, a different approach would have been required to estimate treatment effects with respect to one or more of our performance criteria. Calsyn et al^8^ compared companionship-oriented phone and face-to-face visiting (1.5 hrs per week for 12 weeks) against a no treatment control; finding no effect on life satisfaction in a sample of mostly female (81%) older (mean 77 years) Americans living in the community. However, findings from the Calsyn et al^8^ study may not be applicable to current policy debates given technological advance and social change over the nearly 35 years since this study was conducted. Cheung & Ngan^9^ evaluated the impact of volunteer visiting for frail and socially isolated Hong Kong Chinese. *Increases* in contact intensity and volunteer helpfulness were associated with a significant reduction in worry and increases in community knowledge and social integration but no significant impact on self-reported health. Extrapolating from these results to the effect of treatment *per se* (rather than the effect of *increases* in treatment intensity or quality) in an Australian OWLA population is only possible with a very high level of uncertainty.

For reasons outlined above, results from the literature review were considered uninformative for the purposes of populating the MCDA performance matrix. We therefore populated the performance matrix with uninformative priors and elicited performance ratings from stakeholders and OWLA participants during our small group and one-on-one MCDA sessions based on participants’ expert opinion. Once again, the elicitation of performance ratings followed a script written specifically for this exercise (see Supplementary Materials S2) and we used online electronic forms for data collection; created using *Annalisa* MCDA templates and accessed via the *Elicia* website (see Supplementary Materials S2).

Participants were first provided with a hand-out detailing labels and descriptions of the co-created interventions; followed by moderated discussion to clarify terms and familiarise participants with the co-created interventions. Participants were then shown a brief audio-visual presentation to demonstrate the potential importance of performance ratings in purchasing decisions. At this point, participants were instructed to access the electronic forms for elicitation of the performance matrix and to spend some time mapping from the labels / descriptions of interventions and criteria on the hand-outs to labels of intervention and criteria in the electronic forms.

For elicitation of performance ratings, we anchored the measurement scale at ‘best option’ (100%) and ‘worst option’ (0%). The use of this type of ‘local’ or ‘relative’ scale is appropriate “where criteria will be applied to a one-off decision”; so that performance ratings need only “reflect the best and worst performance observed within the alternatives being evaluated”.^1^  After identifying a best and worst performer on each criteria, participants then rated the performance of other interventions on the 0-100% scale relative to the performance of best and worst performers. Participants completed one column of the performance matrix at a time, with participants rating performance of all interventions on one criteria before moving on to the next column in the performance matrix to rate interventions on the next criteria.

For elicitation of performance ratings, we included a ‘Do Nothing’ option in the set of interventions and elicited performance ratings for ‘Do Nothing’ on each criteria. By ‘Do Nothing’, we mean that participants had access to existing services but not to the set of co-created interventions. Respondents were told to set performance for ‘Do Nothing’ at 0% on the relevant criterion *unless one of the co-created services is expected to make their existing situation worse with respect to that criterion*. This situation (where ‘doing more’ results in lower performance on a relevant criterion) might be expected to arise for finances, where additional services may entail additional out-of-pocket costs.

**Figure S1 – *Annalisa* dashboard for the MCDA exercise**

**
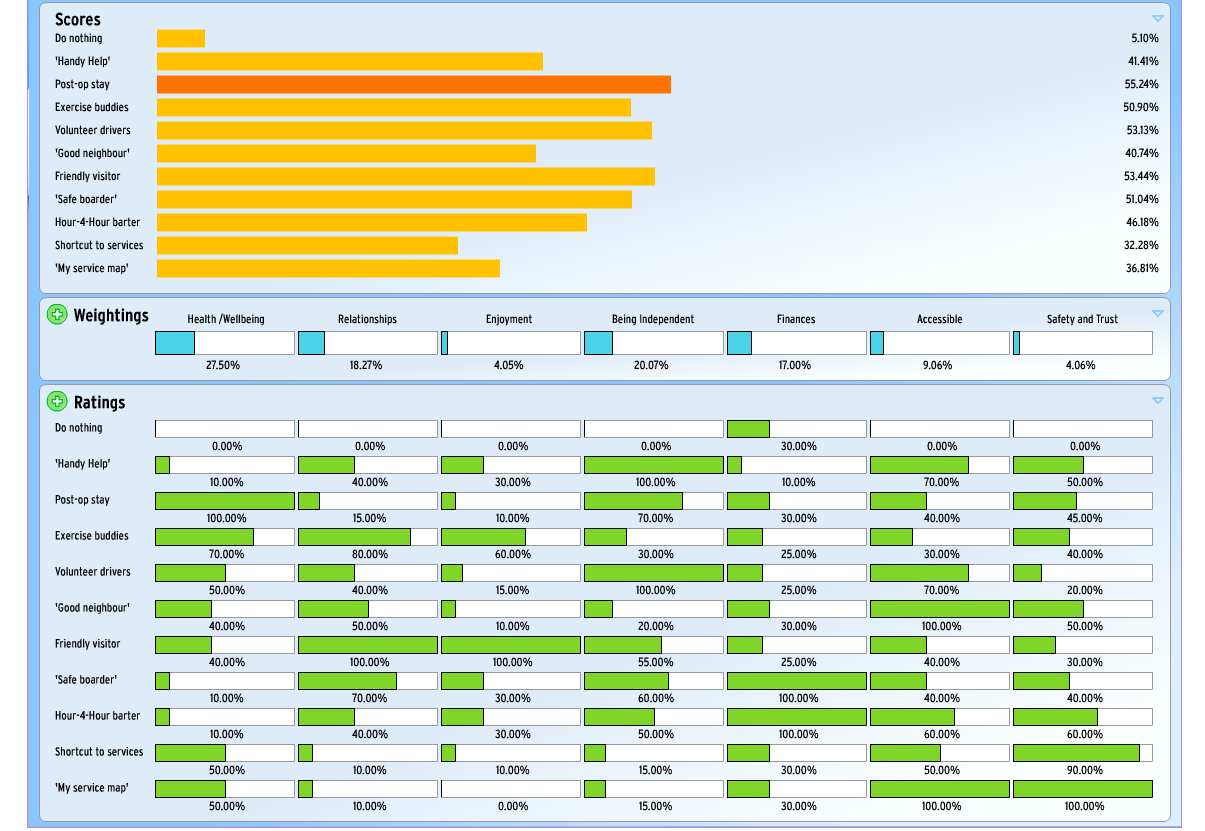
**

***Step 4 – Summarise performance across all criteria***

Performance ratings for each of the co-created interventions and the ‘Do Nothing’ alternative were combined with criteria weights using the *Annalisa* software^10^ to generate intervention scores; summarising each intervention’s performance across all criteria. *Annalisa* calculates intervention scores as a simple weighted sum of performance ratings and criteria weights, with scores ranging from a maximum of 100% (in the event that an intervention receives a performance rating of 100% on *all* criteria) to a minimum of 0% (performance rating of 0% across all criteria). To evaluate the impact of deliberation in updating criteria weights, we calculated scores based on both ‘first-try’ and ‘final’ weights when analysing the data. However, participants were asked to generate intervention scores based only on ‘final’ weights during the small group and one-on-one MCDA sessions. For analysis of weights, ratings and scores, we described heterogeneity / variation in preferences as well as central estimates. Finally, we compared group means for a small number of *a priori* specified groups (OWLA participants versus stakeholder participants).

***Step 5 – Rank from ‘best’ to ‘worst’***

Interventions were rank ordered according to the summary intervention scores calculated at Step 4 of the MCDA exercise. *Annalisa* communicates intervention rankings graphically in a bar chart format, with highest ranked intervention identified with a red bar and ranks of other interventions reflected in the height of an orange bar. Figure S1 above shows the *Annalisa* dashboard for this study, with weights and ratings entered for a hypothetical participant, and with scores and ranks calculated based on these weights and ratings. After generating scores and ranks, participants were asked to consider whether global or holistic assessment of the ‘value’ of each intervention to OWLA would produce a similar set of scores and ranks. All sessions concluded with a short moderated discussion of possible reasons for any differences between a global or holistic assessment of value and scores / ranks generated from the MCDA exercise.

**REFERENCES**

1. Peacock S, Mitton C, Bate A, McCoy B, Donaldson C. Overcoming barriers to priority setting using interdisciplinary methods. *Health Policy*. 2009; **92**: 124-32.
2. Youngkong S, Teerawattananon Y, Tantivess S, Baltussen R. Multi-criteria decision analysis for setting priorities on HIV/AIDS interventions in Thailand. *Health Res Policy Syst*. 2012; **10**: 6.
3. Marsh K, IJzerman M, Thokala P, Baltussen R, Boysen M, Kalo Z, *et al*. Multiple Criteria Decision Analysis for Health Care Decision Making--Emerging Good Practices: Report 2 of the ISPOR MCDA Emerging Good Practices Task Force. *Value Health*. 2016; **19**: 125-37.
4. Spinks J, Mortimer D. Lost in the crowd? Using eye-tracking to investigate the effect of complexity on attribute non-attendance in discrete choice experiments. *BMC Med Inform Decis Mak*. 2016; **16**: 14.
5. Dickins M, Lowthian J, Ogrin R, Enticott J, Mortimer D, Browning C, *et al.* The effects of interventions for addressing barriers and enablers of access to care to improve health and wellbeing in older people living alone: a systematic review. *PROSPERO*, 2017; **CRD42017053298**. Available from: <http://www.crd.york.ac.uk/PROSPERO/display_record.php?ID=CRD42017053298>
6. Feldman S, Radermacher H. Time of our lives? Building opportunity and capacity for the economic and social participation of older Australian women. Melbourne: Lord Mayor’s Charitable Foundation; 2016.
7. Andrews G, Gavin N, Begley S, Brodie D*.* Assisting friendships, combating loneliness: Users' views on a ‘befriending’ scheme. *Ageing and Society*, 2003; **23**: 349-362.
8. Calsyn R, Munson M, Peaco D, Kupferberg J, Jackson J*.* A comparison of the effectiveness of various approaches to visiting isolated community elderly. *Journal of Gerontological Social Work*, 1984; **7**: 29-42.
9. Cheung C-K, Ngan M-H. Contributions of Volunteer Networking to Isolated Seniors in Hong Kong. *Journal of Gerontological Social Work*, 2000; **33**: 79-100.
10. Dowie J, Kjer Kaltoft M, Salkeld G, Cunich M. Towards generic online multicriteria decision support in patient-centred health care. *Health Expect*. 2015; **18**: 689-702.
