# Supplementary material for: Using co‐creation and multi‐criteria decision analysis to close service gaps for underserved populations
Source: Health Expect. 2019 Jun 11;22(5):1058–68. doi: 10.1111/hex.12923 (PMC6803401; doi:10.1111/hex.12923)
Supplement: Supplementary file 2 [file HEX-22-1058-s002.docx]

## Appendix S2: RUNNING SHEET, SCRIPT AND MATERIALS FOR MCDA EXERCISE

***[RO: Welcome & introductions]***

***..***

***[DM: Background ~ 5 mins]***

Thanks for coming.

Let me tell you a little bit about what we have in mind for today.

The team has conducted lots of interviews and looked at the research and come up with seven ‘criteria’ which seem to be important when people decide what makes a good service good.

As with all decisions, it’s quite likely that some criteria will be very important to some people but less important to others. And, in fact, we might expect the importance of different criteria to depend upon your background and circumstances.

For this reason, we will ask each of you to score our seven criteria based on how important they are to you when deciding what makes a good service good. We will call these scores ‘importance weightings’.

We have two different groups here today. Some of you are here from organisations that help older women. Your job is – as best you can - to represent the views of the older women that your organisation helps. We encourage you to use your personal and professional experience to decide upon your importance weightings. For everyone else, we’re looking for your own personal views.

Your importance weightings are central to this exercise so we will ask you to do this bit twice. The first time, we’ll ask you to weight the seven criteria without discussing your views with others here today. There are no right or wrong answers and we’ll take you through the process one step at a time. We’ll then discuss what you all thought about the importance of our seven criteria and you’ll get a chance to hear different views and change your importance weightings if you want to.

Working through this process will be a big part of the day.

We have some other jobs on our to-do list as well.

**We also** want to rank the services that we’ve been talking about in the other forums you’ve attended, running from best to worst. We’ll do this based on (i) the importance of each of our seven criteria, and (ii) how well each service performs across these seven criteria.

**Finally** we’ll hopefully have time to discuss whether you agree with this best to worst ranking, whether you’d like to change it, and why?

To do all this, we’re going to be using a program on the computer. You’ll get help using this program from the friendly faces you see around the room. You know most of these people from the other forums you’ve attended.

Any questions about what we’re going to do today?

I’d like to show you a short video now leading in to our first task for the day. Before I do that can I ask a mundane question: what is important to you when buying a fridge? What criteria do you use to choose a fridge?

*[DM to moderate short discussion, 2-3 responses]*

Okay, let’s look at the video…

*[DM: Play Mini-Mooc up to 43sec mark* [*https://www.youtube.com/watch?v=9gXNG34yVDU&feature=youtu.be*](https://www.youtube.com/watch?v=9gXNG34yVDU&feature=youtu.be) *]*

***[Elicit individual criteria weightings: Bracket 1 ~ 30 mins]***

Now you’re going to get a chance to find your own criteria weightings. Not for fridges, we’re going to instead find criteria weightings for health and community services.

First, let’s look at our seven criteria.

*[DM: Show slide of cheat sheet with criteria labels and descriptions]*

*[HELPERS: Hand-out a copy of cheat sheet to all OWLA and stakeholders]*

*
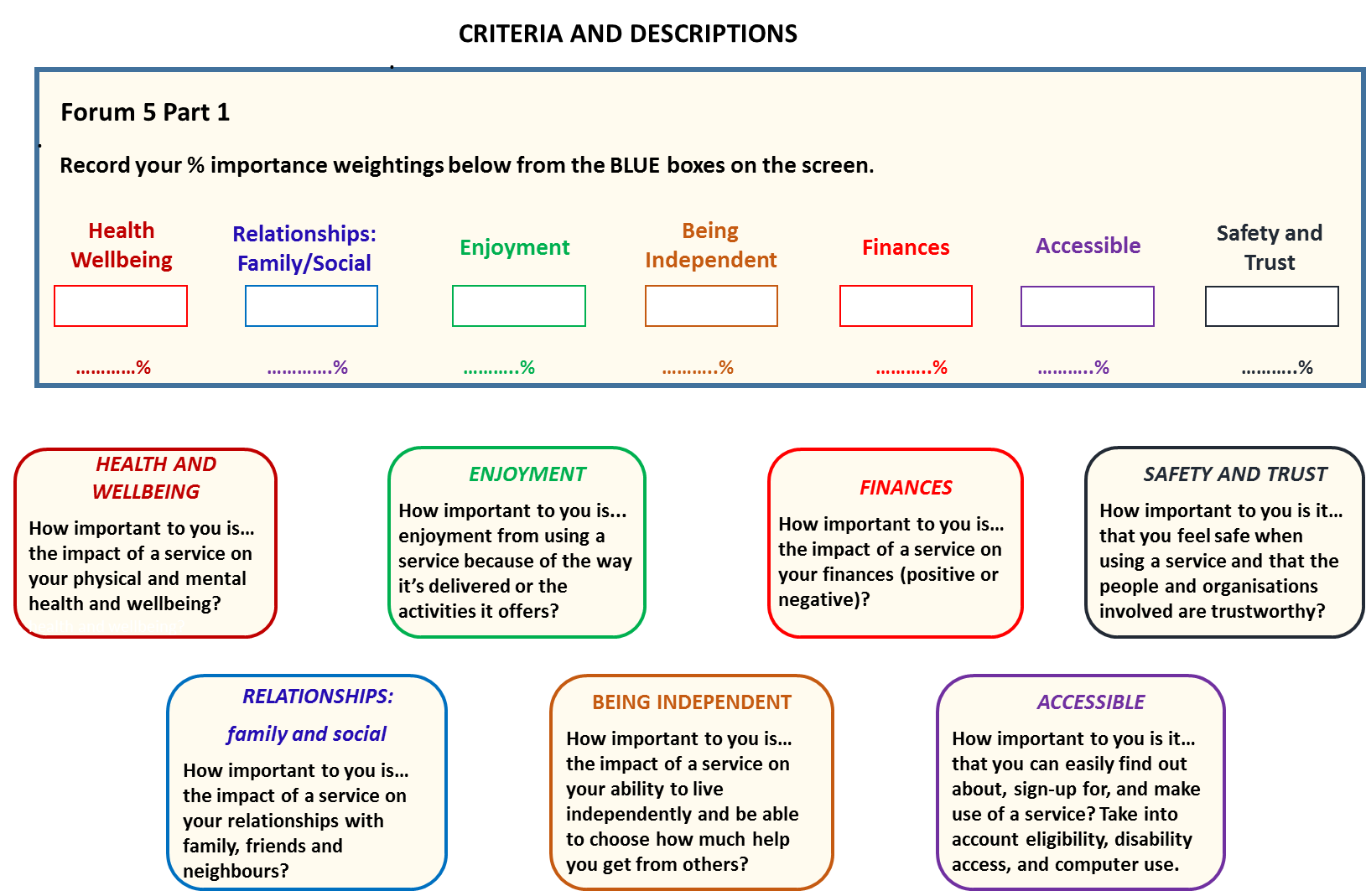
*

*[DM: Talk through the criteria labels and descriptions]*

Now we get to use our fancy software.

If you ‘wake-up’ the lap-top in front of you, you’ll see a screen that looks a bit like this.

Our helpers are on-hand to help you. Please call out if your computer won’t wake up.

*[DM: Show Elicia entry screen for survey]*


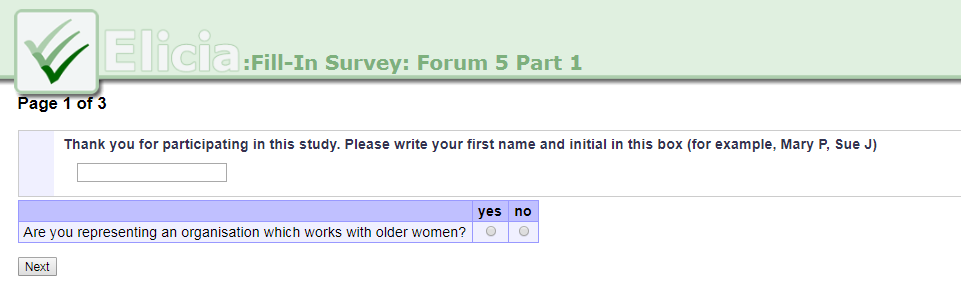


We’d like you to enter your first name and initial in the box on this screen. This is just so that we can make sure that we don’t mix up different people’s opinions at different stages of today’s exercise. We’ll never identify who said what when we report our results.

Once you’ve entered your name and answered the question, we’d like you to click the ‘NEXT’ button at the left of the screen.

Once again, please call out if you are having any trouble at all.

Now you should see our seven criteria on your screen. Each with a box underneath. You’ll see that we have 100% to share between our criteria. We could have put the 100% in any one of the criteria boxes or split it across boxes. Here, we’ve taken the simplest approach and put the full 100% in the first box.


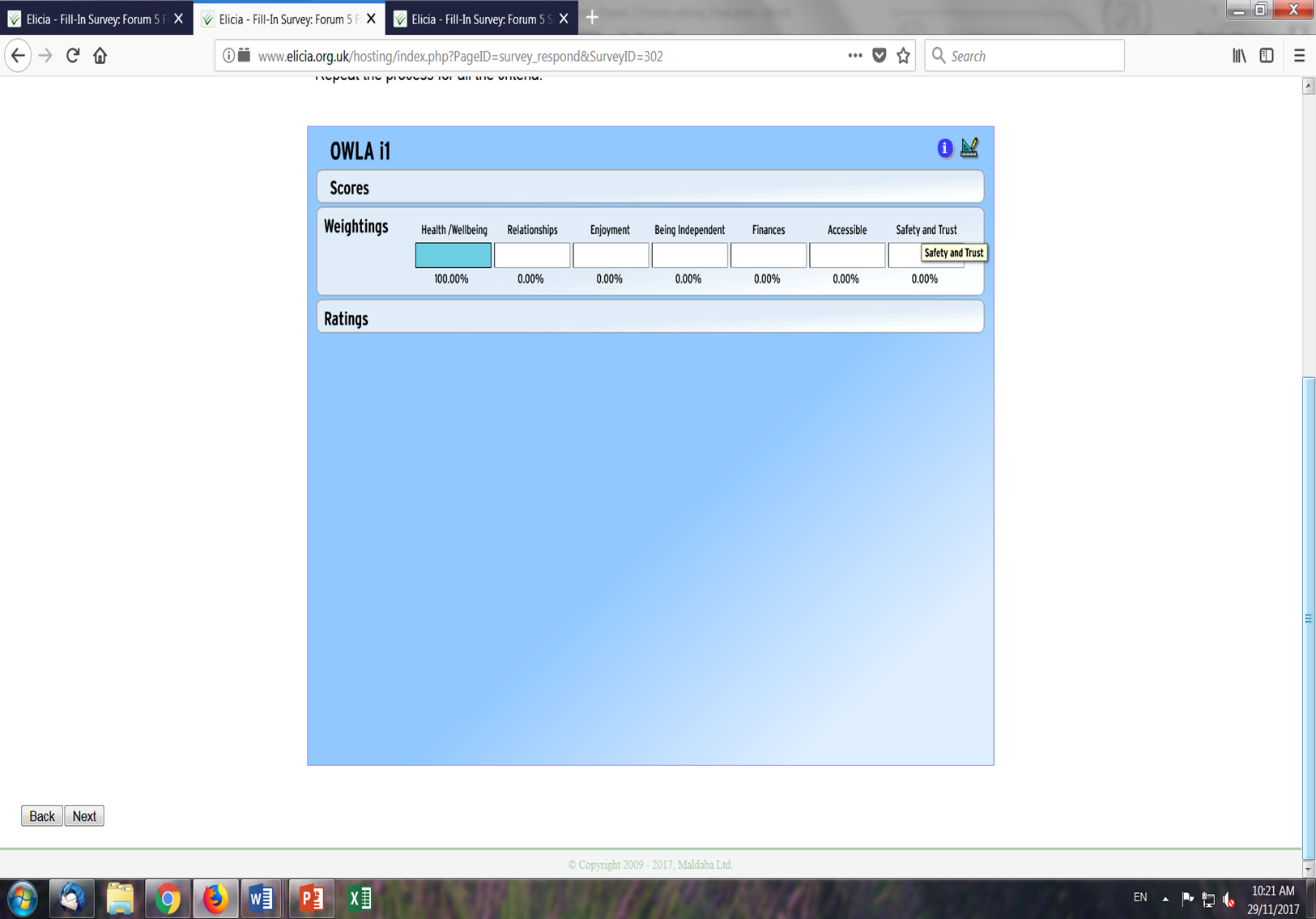


***First,*** I’d like you all to work out which is the most important criteria to you (or the older women your organisation helps). Then use your mouse to left-click and hold down anywhere in the box for that criteria, then drag all the way to the right until the box fills with blue and you see ‘100%’ under the box.

Rajna and Marissa and Georgina and Angelo are on hand to help if you are having trouble with the mouse or the slider.

Have we all done that?

***Now,*** I want you to think about the second most important criteria and - if this criteria is at all important to you - I want you to put some blue in the box for this criteria. Again, use your mouse to left-click and hold down anywhere in the box for that criteria, then drag all the way to the right until the box has the right amount of blue. The amount of blue – the weight for this criteria – should show the relative importance of your first and second criteria.

If, for example, the second criteria is half as important as the first, then you should increase the weight on the second criteria until it is half that of the first criteria.

This would be at 33.33% and 66.67% - NOT at 50% and 100% - because all our weightings must add up to 100%.

Have we all done that?

***Next,*** find the third most important criteria to you and put some blue in its box if it is important to you.

The amount of blue should show the relative importance to you of your second and third criteria.

If, for example, your third criteria is equally as important to you as your second criteria, then you would end up with 25% for the third most important, 25% for the second most important and 50% for the most important.

Have we all done that?

***Okay,*** let’s run through that same process for the fourth, fifth, sixth and seventh most important ‘importance’ criteria. Once again, please call out if you need some help with the computer.

Phew, we’re nearly done with our first task. Just a couple more steps to go.

Remember the sheet we handed out earlier in the session. It looks like this…

*[DM: Show slide of cheat sheet with criteria labels and descriptions]*

*
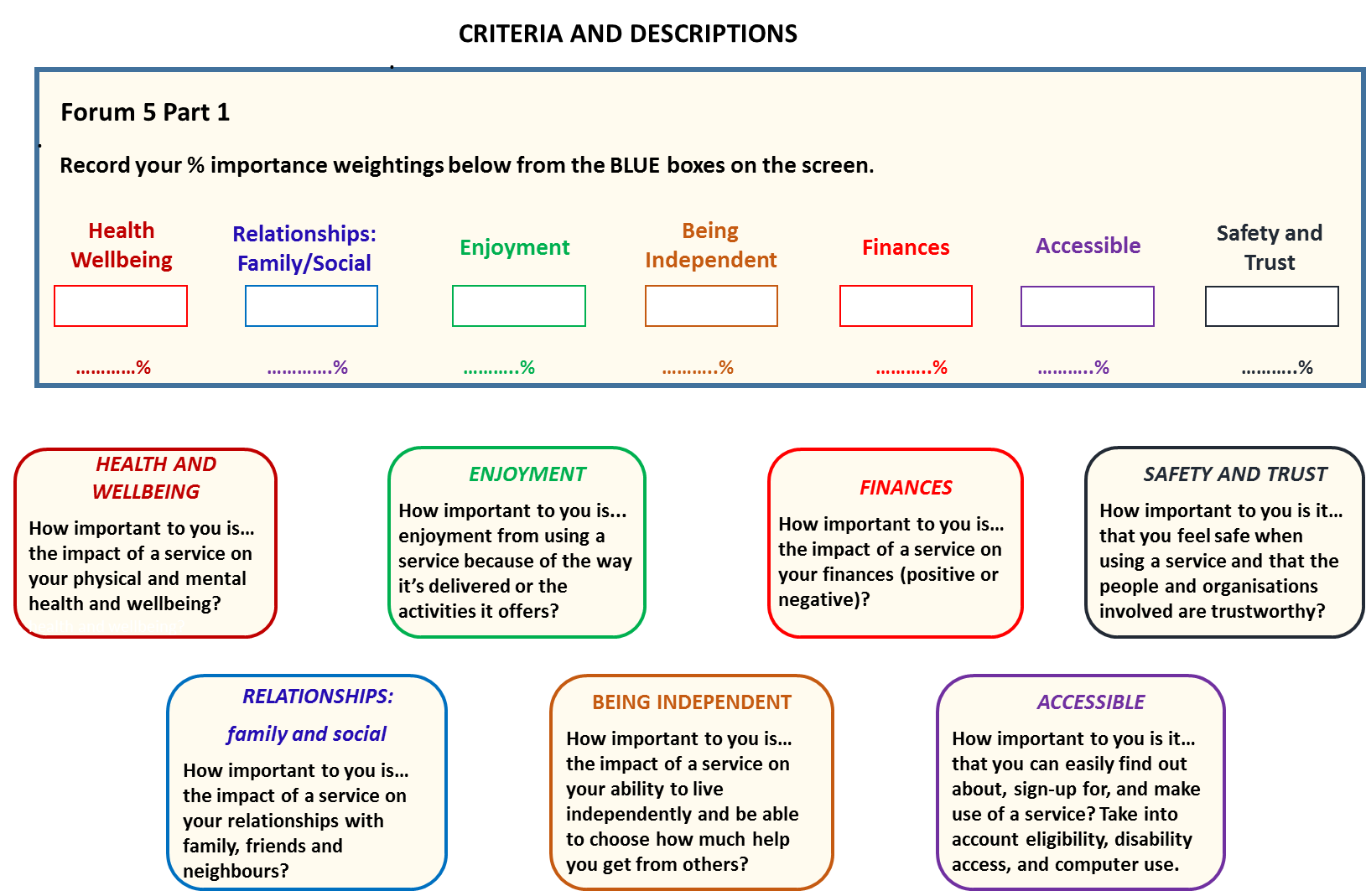
*

I just want you to write down the numbers from your screen – your % weightings – on to the sheet of paper in the ‘First Try’ box, the top box. You don’t have to do any colouring in on the piece of paper, just the numbers will do fine. Now write you first name and initial in the top right corner of the piece of paper.

Have we all done that? Okay, find a safe spot for your sheet. You’ll need it in a little while.

Hooray, we’ve got our criteria weightings!!!

Now, I’d like you to click on the ‘NEXT’ button and then the ‘SUBMIT YOUR ANSWERS’ button on the next screen and I’m going to hand you over to Angelo for a bit of a chat about what we’ve just done.

***[Break: ~ 10 mins]***

*[DM: Download data from Elicia and calculate average weightings for all participants, stakeholders and consumers, create slides for room average and group averages]*

*[RO et al: Encourage participants to eat catering, get a coffee/tea, helpers to chat with participants for first couple of minutes]*

*[AI: Until DM ready with data, AI to moderate discussion about which criteria respondents thought were most important and why]*

**[Elicit deliberative weightings: Second Try ~ 10 mins]**

*[DM: Show slide for room and group averages]*

Now, let’s see what you said about our seven criteria.

*[DM: Report back to the room with average weightings for all participants, average weightings for stakeholder participants, and average weightings for older women living alone]*

*[DM: Short moderated discussion of differences between average and individual weightings, differences between group averages, and reasons for any such differences]*

Okay, so let’s finalise our importance weightings. We’ve had a chance to hear what others thought.

And you’ve now got a chance to make any changes. Don’t feel pressured to make a change just for the sake of it. Only make a change if you think that the weightings from your first try don’t really reflect what YOU think NOW after hearing the discussion and seeing results from the first try.

Let’s wake up our computers again.

Can you now find the page named **‘FORUM 5 Second Try’** that’s open on your computer? If the file isn’t already open look for a tab near the top of your computer.

Please open this page or tab. It should look like this…

*[DM: Show slide for Elicia entry page for Second Try]*


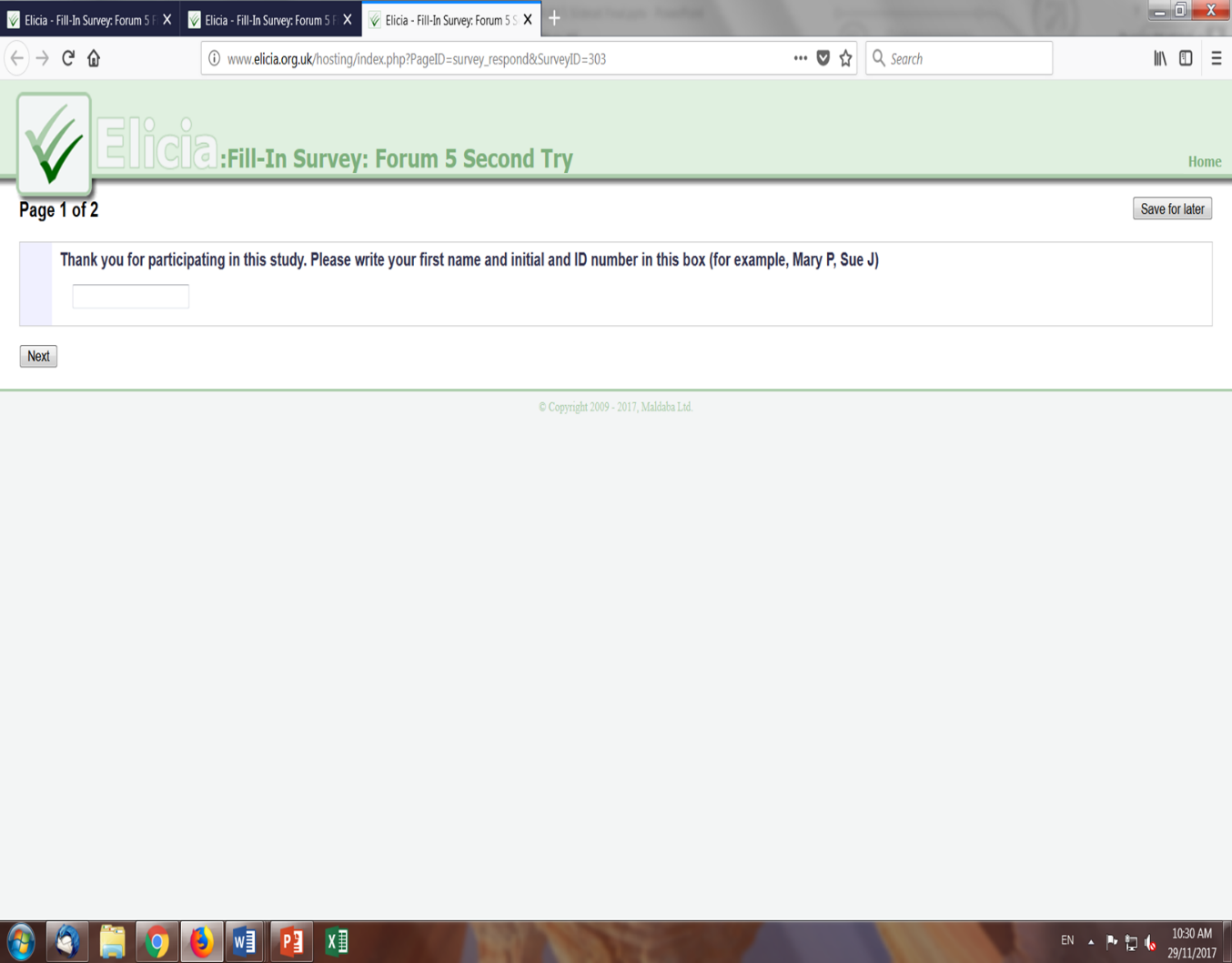


Once you see this screen again, please enter your first name and initial again and then click on the ‘NEXT’ button at the left of the page.

Now we can see our criteria again but we are back at our starting point with the full 100% in the first box.


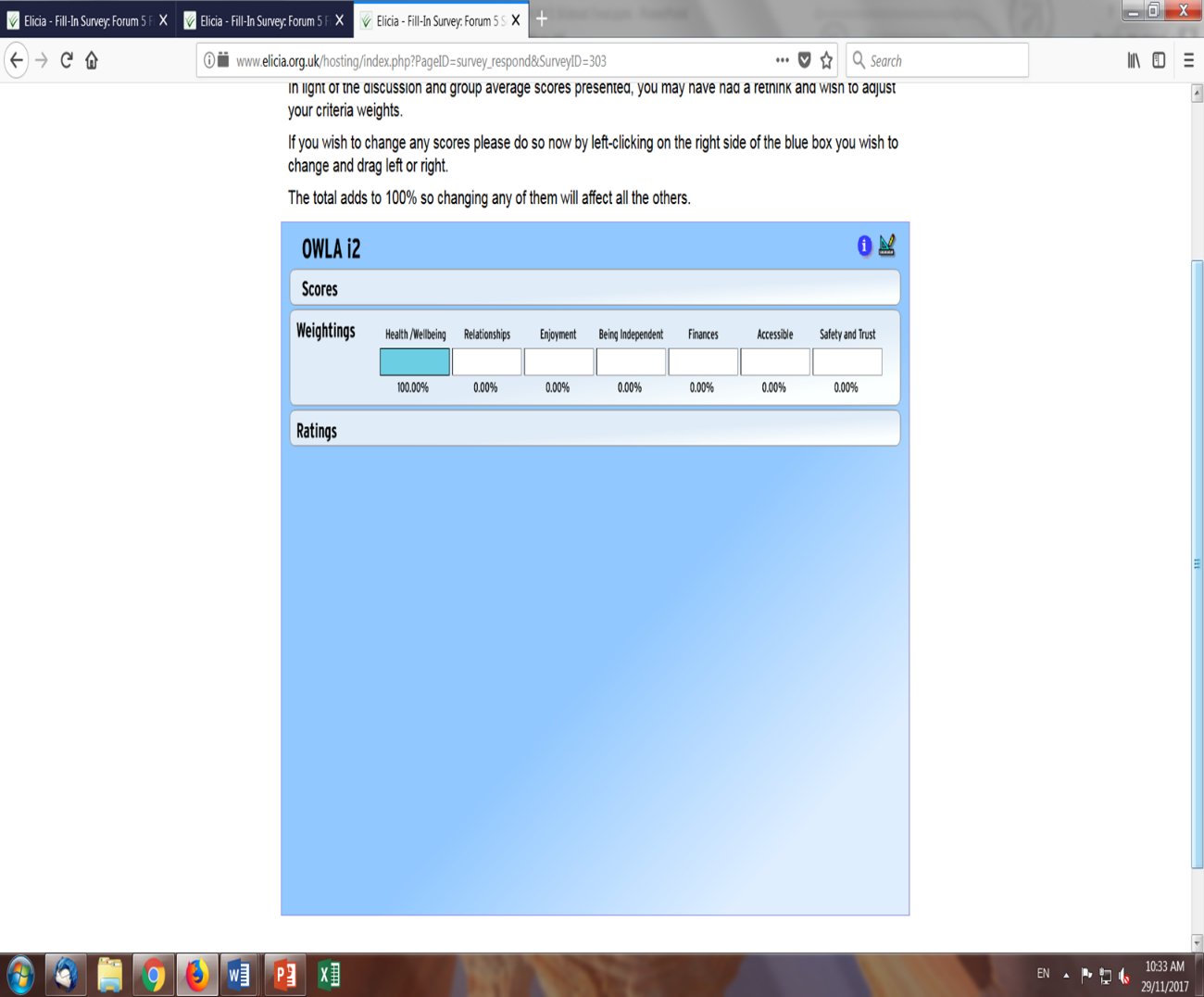


I want you to take the hardcopy sheet that you used to record the weightings from your first try.

Can you please type the weightings from your first try into the box for each of our criteria?

Have we all done that?

***Now***, can you think about whether these weightings still look right? Do you want to change anything about them? If so, now is the time.

Remember, you can always just type in the weightings if you are having trouble with the mouse. Let us know if you need any help.

[Allow 5 minutes]

Have we all done that? Excellent!

*[HELPERS: Hand-out the ‘SECOND TRY’ work/cheat sheet to all OWLA and stakeholders]*

***Now,*** our helpers are handing out a worksheet for you to enter your weightings.


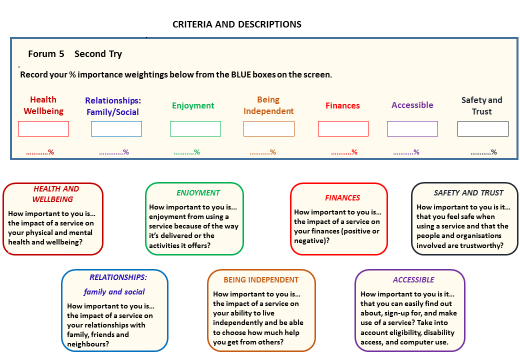


Can you please write down the numbers from your screen – your % weightings – in the ‘Second Try’ box? Again, you don’t have to do any colouring in on the piece of paper, we just want the numbers. Now write you first name and initial in the top right corner of the piece of paper.

Have we all done that? Okay, find a safe spot for your sheet. You’ll need it in a little while.

Now, I’d like you to click on the ‘NEXT’ button at the bottom of the screen and then the ‘SUBMIT YOUR ANSWERS’ button on the next screen.

Now we can move on to the job of rating the performance of the services that we’ve been talking about in the other forums you’ve attended.

***[Performance ratings and revision of summary scores and rankings: FINAL TRY ~ 45 mins]***

Let’s have another look at these services…

*[DM: Show slide of* ***SERVICES LIST and descriptions****]*

*
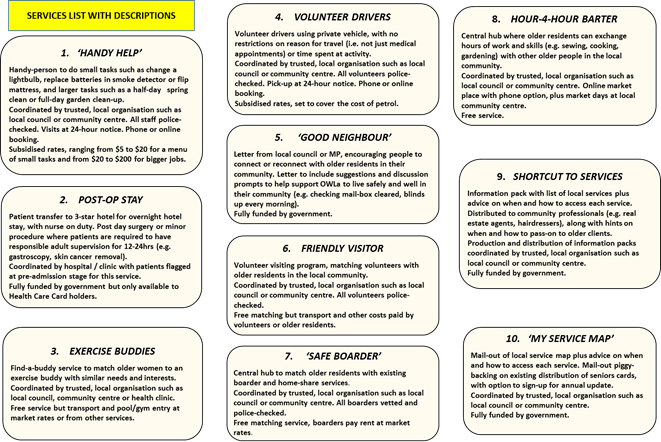
*

For this exercise, we’ve brought together what was said in the forums and used this information to ‘nail down’ the key features of the 10 services you see here.

Please take a minute to read through the description for each of these options. Each has a short label that we’ll use for the rest of the exercise and that you’ll see in the computer program.

*[HELPERS: Hand-out a copy of cheat sheet to all OWLA and stakeholders]*

*[DM: Talk through the option labels and descriptions]*

What if we could only provide one or two of these services? It might be quite a difficult decision. They’re all quite different and it might be difficult to keep track of these differences.

Let’s think a bit more about this type of choice using fridges as an example. The importance of different criteria for choosing a fridge is key BUT we also need to know how each fridge performs on these criteria.

Let’s look at a bit more of our video…

*[DM: Play Mini-Mooc up to 1:14 mark* [*https://www.youtube.com/watch?v=9gXNG34yVDU&feature=youtu.be*](https://www.youtube.com/watch?v=9gXNG34yVDU&feature=youtu.be) *]*

Now we’ve seen how to rate the performance of fridges. We’re going to run through the same process to rate the performance of our health and community services against each of our seven criteria.

This is the most important part of the whole exercise so freshen up, have a drink or take a 5 minute toilet break.

[Allow 5 minutes]

Let’s wake up our computers again.

Can you now find the tab named ‘**FORUM 5 Final Try’**?

Please open this tab and you should find yourself back on our front page. Please enter your first name and initial again and then click on the ‘NEXT’ button at the left of the page.

*[DM: Show front page for final try]*


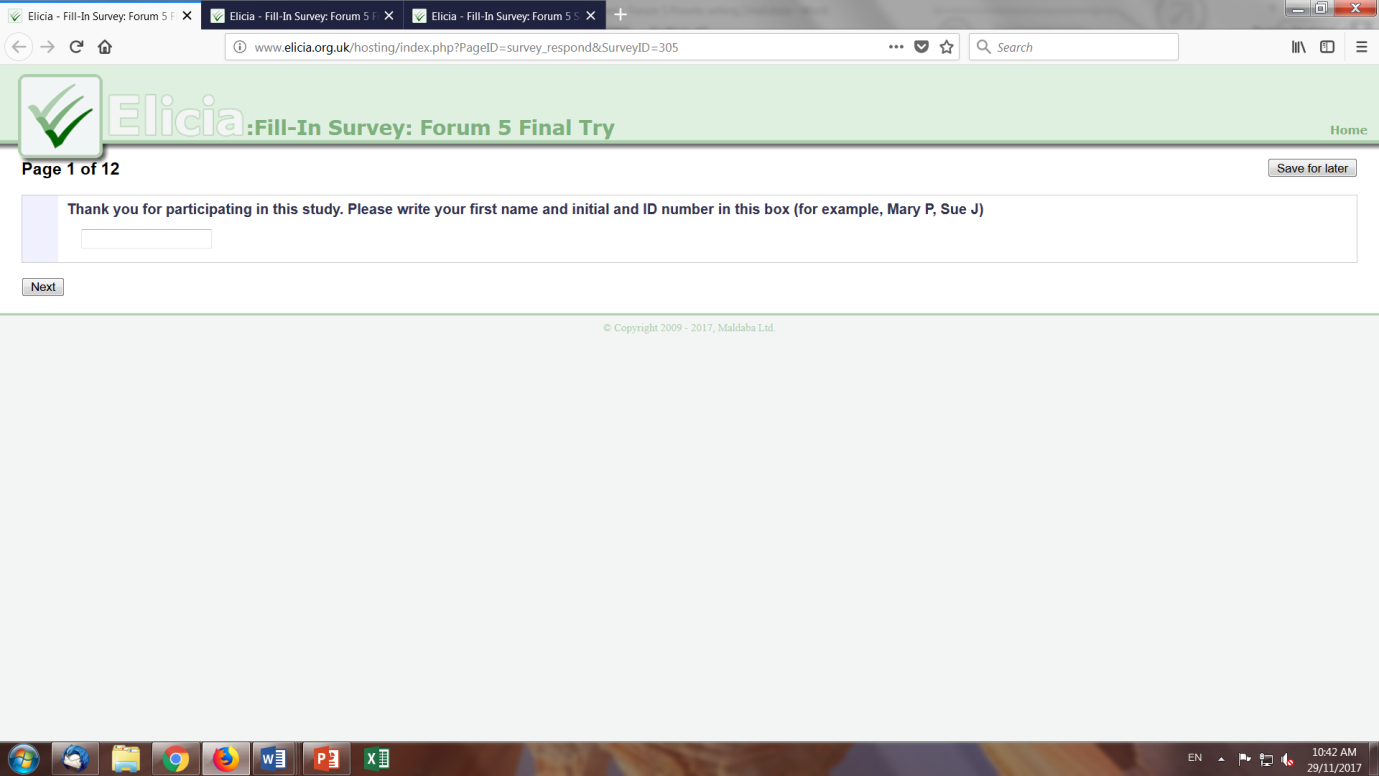


*[DM: Show Annalisa screen for final try]*


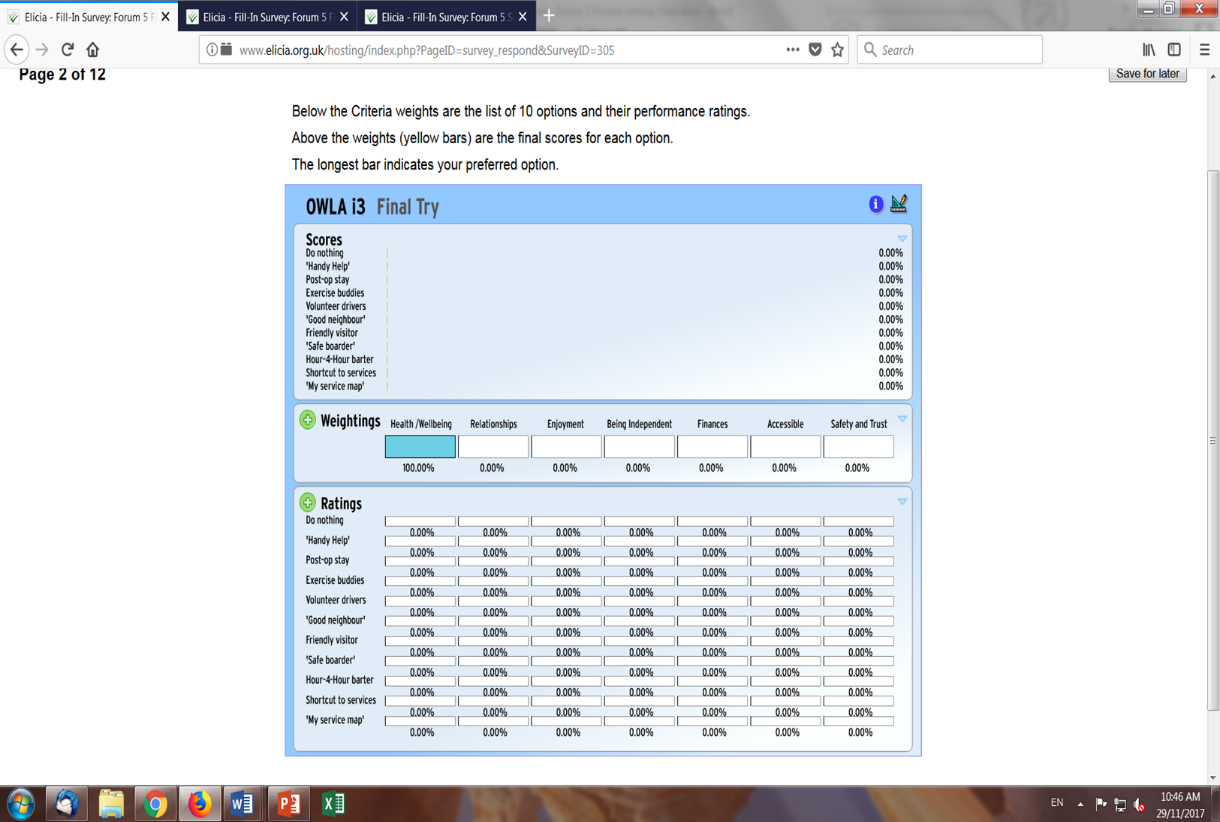


Now you should see the familiar ‘weightings’ box but you’ll also see a ‘ratings’ box below the weightings box and a ‘scores’ box above. For the moment, we’re going to concentrate on the ratings box and I want you to hide the ‘scores’ box by clicking on the blue upside down triangle in the top right-hand corner.

Once you’ve done this, your screen should be a bit easier to read and should look like this…

*[DM: Show Annalisa screen for final try with Scores box hidden]*


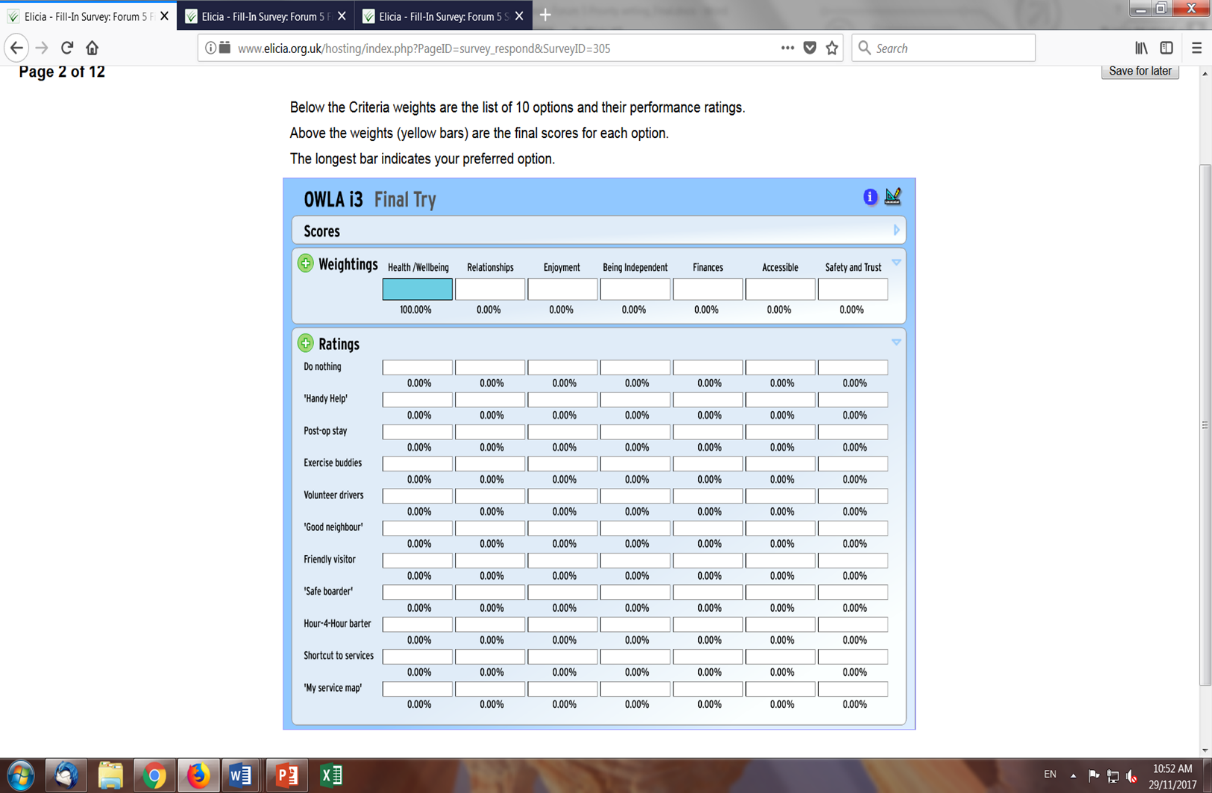


Okay, so let’s look at the ratings box.

The ratings box shows how each of our services performs on each criteria. At the moment, we’ve set the ratings to zero for all services and for all criteria.

This would mean that none of our services are very good and we’d actually be saying that all services are as bad as the worst performer on each criteria.

We’re hoping this isn’t true.

If a service was the best performer on a criteria, then it would have its box for that criteria filled with green and its rating for that criteria would be 100%.

Please note that each ratings box can range from 0-100%. The rows don’t have to add up to 100%. Nor do the columns.

**First,** we’re going to work **ONLY WITH THE FIRST COLUMN** and rate the performance of our services on Health & Wellbeing. Remember, Health & Wellbeing is… *the impact of a service on your physical and mental health and wellbeing*.

I want you to read through our list of services and work out which service you believe has the best performance on Health & Wellbeing and which has the worst.

You’ll see that we’ve added ‘do nothing’ to the list of options in our ratings box. By ‘do nothing’, we mean that you have access to other existing services but not to the services we’ve listed here and that we’ve been thinking about adding to what is currently available.

Unless you think that one of our ‘added extra’ services would make your Health & Wellbeing worse, then you’d probably nominate ‘do nothing’ as the worst performer on Health & Wellbeing.

Once you’ve found what you believe is the best service, I want you to change its rating on Health & Wellbeing – remember we are working only down the first column – to 100%.

Leave the worst service in terms of Health & Wellbeing at 0% but then work through and rate performance on Health & Wellbeing for all other services (again, working down the first column in the ratings box).

If a service is no better than ‘do nothing’ on Health & Wellbeing, then it should get 0%.

If a service is just as good as your best performer, then it should get 100%.

For other services, give them a rating of between 0 and 100% that reflects their performance on Health & Wellbeing.

**Second,** we’re going to work down the second column… rating the performance of our services on Relationships. Remember, Relationships is… *the impact of a service on your relationships with family, friends and neighbours*.

I want you to read through our list of services and work out which service you believe has the best performance on Relationships and which has the worst.

Once you’ve found the best performer, I want you to change its rating on Relationships (second column in the ratings box) to 100%.

Leave the worst performer at 0% but then work through and rate performance on Relationships for all other services (again, working down the second column in the ratings box).

Services that aren’t the best and that aren’t the worst should get a rating of between 0 and 100%, with better performance on Relationships reflected in higher ratings.

**Third,** let’s run through the same process for Enjoyment. By enjoyment we mean, *…enjoyment from using a service because of the way it’s delivered or the activities it offers.*

Read through our list of services and work out which service you believe has the best performance on Enjoyment and which has the worst.

Once you’ve found the best performer, I want you to change its rating on Enjoyment (third column in the ratings box) to 100%.

Leave the worst performer at 0% but then work through and rate performance on Enjoyment for all other services (again, working down the third column in the ratings box).

**Now**, let’s do the same for our remaining criteria.

Set your best performer on each criteria to 100% and your worst to 0%, then choose a rating between 0 and 100% for each of the other services.

[Allow 10 minutes]

Have we all done that? Across all services and all criteria?

Excellent!

**Now** let’s look at what happens when we combine our importance weightings with our performance ratings.

To do this, we need to enter our ‘SECOND TRY’ weightings into the weighting box.

Have we all done that?

Excellent! Now we have everything we need to decide which service to provide.

We can find this directly from our ‘scores’. We need to unhide the ‘scores’ box by clicking on the little blue arrow again.

Can you see the scores?

The service with the highest score should be YOUR preferred option.

Now, this might not be the result that you were expecting. I don’t want you to change your weightings or performance ratings at this point but we do want to know whether you agree or disagree with what Annalisa is telling us.

*[DM: Short moderated discussion of agreement / disagreement]*

Okay, thanks for your feedback. We’ll take note of these comments in deciding where to go from here.

Now I’d like you to click on the ‘NEXT’ button at the bottom of the screen.

You should then see some questions about your background and your experience with the type of services we’re been looking at today. Can you please complete these questions and then click NEXT / SUBMIT and we’re done.

Thanks for all your hard work on this. Lots to think about and I hope you’re not too exhausted….
